# Efficacy and safety of orforglipron in adults with body mass index of 27 to less than 30 kg/m^2^ and adiposity-related complications: A post hoc analysis of the ATTAIN-1 trial

**DOI:** 10.1016/j.obpill.2026.100346

**Published:** 2026-10-14

**Authors:** Jaime P. Almandoz, Alexander Dimitri Miras, Matthias Blüher, Pedro Orozco Del Pino, Donna Mojdami, Rachel L. Batterham, Kevin Trice, Benjamin Goebel, Fatih Tangi

**Affiliations:** a Department of Internal Medicine, Division of Endocrinology, University of Texas Southwestern Medical Center, Dallas, TX, USA; b School of Medicine, Faculty of Life & Health Sciences, Ulster University, UK; c Helmholtz Institute for Metabolic, Obesity and Vascular Research (HI-MAG), Helmholtz Center Munich, Leipzig University and University of Leipzig Medical Center, Leipzig, Germany; d University of Leipzig Medical Center, Leipzig, Germany; e Eli Lilly and Company, Indianapolis, IN, USA

**Keywords:** Cardiometabolic risk, Orforglipron, Adiposity-related complication, Weight reduction, Quality of life, Overweight or obesity

## Abstract

**Background:**

Orforglipron, an oral small-molecule glucagon-like peptide-1 receptor agonist, improved body weight and cardiometabolic parameters in the phase 3 ATTAIN-1 trial, but its efficacy and safety in adults with body mass index (BMI) of ≥27 to <30 kg/m^2^ and adiposity-related complications have not been characterized. Adults with overweight and an adiposity-related complication are at substantial cardiometabolic risk, and obesity medications are recommended for this group.

**Methods:**

This was a post-hoc analysis of the ATTAIN-1 trial (NCT05869903), a phase 3, randomized, double-blind, placebo-controlled trial in which adults without diabetes and with obesity or overweight were randomized 3:3:3:4 to once-daily oral orforglipron 5.5, 9, or 17.2 mg or placebo for 72 weeks. The present analysis included the subset of randomized participants with BMI ≥27 to <30 kg/m^2^ and ≥1 adiposity-related complication. Endpoints included change from baseline to Week 72 the percent change in body weight, BMI, categorical weight-loss, waist circumference, lipids, patient-reported outcomes, and safety. Effect estimates are reported using efficacy and treatment-regimen estimands. Efficacy endpoints were also reported per sex subgroup.

**Results:**

This analysis included 260 participants (orforglipron 5.5 mg: 56; 9 mg: 64; 17.2 mg: 62; placebo: 78). At Week 72, orforglipron was associated with greater mean percent body weight reduction (17.2 mg: −13.5%) versus placebo (−0.3%) (nominal p < 0.001). Females experienced up to −18.8% mean body weight reduction and males up to −10.0%. Orforglipron was associated with improvements in waist circumference, triglycerides, and low-density lipoprotein cholesterol, as well as improvements in patient-reported outcomes. Safety profile was consistent with previous trials with predominantly mild-to-moderate gastrointestinal events.

**Conclusion:**

In this post hoc analysis of ATTAIN-1, participants with BMI ≥27 to <30 kg/m^2^ and ≥1 adiposity-related complication, orforglipron was associated with improvements in anthropometric and cardiometabolic parameters, with a safety profile broadly consistent with the overall ATTAIN-1 trial (ClinicalTrials.gov identifier: NCT05869903).

## Highlights

• Orforglipron was associated with greater mean weight loss (17.2 mg: −13.5%) in patients with BMI below 30 kg/m^2^ and adiposity-related complications, with females reaching up to 18.8% mean weight reduction and males reaching up to 10.0% mean weight reduction; nominal p < 0.001 versus placebo.
• Clinically meaningful improvements were observed in BMI, waist circumference, and cardiometabolic parameters.
• Over half of participants treated with orforglipron 17.2 mg reached BMI <25 kg/m^2^.
• Safety and gastrointestinal tolerability in this specific group were generally consistent with other GLP-1 receptor agonist medicines.
• Data support potential benefits for patients with BMI<30 kg/m^2^, but are limited by a small, predominantly Asian subgroup.

## Graphical abstract

**Figure:**
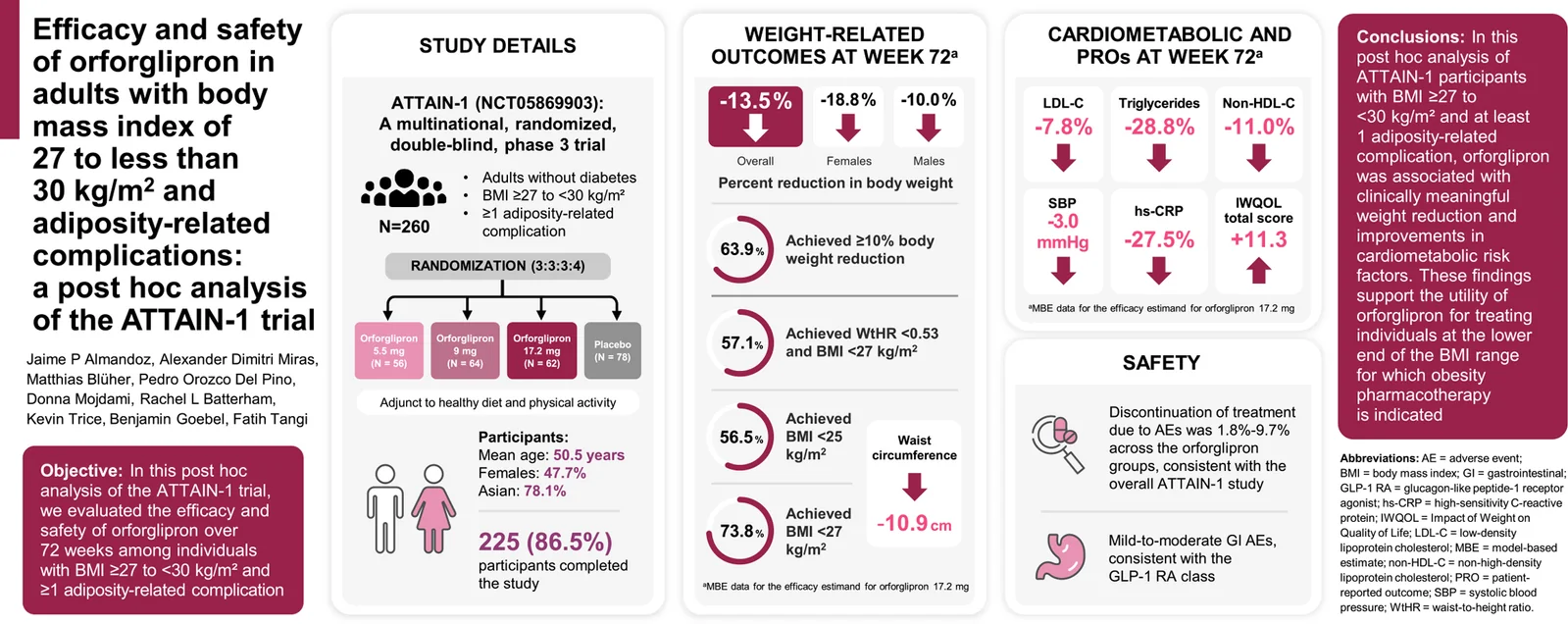

## 1 Introduction

Obesity is a chronic, progressive, relapsing disease that drives a wide range of cardiometabolic, functional, and psychosocial complications, affecting physical health, mental well-being, and quality of life [1], [2]. Adiposity-related complications, although tending to increase with adiposity, are not confined to specific body mass index (BMI) thresholds [2]. Indeed, a substantial proportion of adults classified as having overweight (BMI 25–29.9 kg/m^2^) already have adiposity-related complications, such as hypertension, dyslipidemia, obstructive sleep apnea (OSA), or dysglycemia that contribute to impaired life expectancy and quality of life [3], [4]. Moreover, people classified as having overweight are at a higher risk of developing many of the common complications of obesity compared to those with a BMI <25 kg/m^2^, including cardiometabolic diseases, osteoarthritis, and metabolic dysfunction-associated steatotic liver disease (MASLD) [5], [6], [7]. Notably, the Asian population has been recognized to develop adiposity-related complications at lower BMI levels; accordingly, the World Health Organization (WHO) recommends lower BMI thresholds for overweight (≥23 kg/m^2^) and obesity (≥27.5 kg/m^2^) among Asian adults [8].

While multiple guidelines recommend the use of obesity medications for people with overweight and at risk of developing adiposity-related complications (i.e. BMI <30 kg/m^2^) [9], [10], [11], there is a paucity of clinical data focused on the BMI 25–29.9 kg/m^2^ subgroup with adiposity-related complications, and obesity medications often remain underutilized until advanced stages of obesity [12]. Further, the recent American Association of Clinical Endocrinology (AACE) and American Diabetes Association (ADA) guidelines emphasize the presence and severity of adiposity-related complications when establishing treatment goals and selecting pharmacotherapy, rather than relying on BMI alone [10], [13].

Orforglipron is a once-daily, oral non-peptide small-molecule glucagon-like peptide-1 (GLP-1) receptor agonist (RA) that can be taken without restrictions on food or water [14]. Orforglipron is approved for the treatment of obesity or overweight with obesity-related medical conditions [15]. In the phase 3 ATTAIN-1 trial of adults with obesity or overweight with adiposity-related complication and without diabetes, orforglipron 5.5, 9, and 17.2 mg (equivalent to 6, 12, and 36 mg, respectively [16]) reduced body weight by 7.8%, 9.3%, and 12.4%, respectively, versus 0.9% with placebo at Week 72 (efficacy estimand), and improved cardiometabolic parameters like waist circumference, systolic blood pressure, triglycerides, and non-high-density lipoprotein (HDL) cholesterol; adverse events (AEs) were predominantly gastrointestinal and mostly mild-to-moderate, consistent with other GLP-1 medicines [14].

Cardiometabolic risk and BMI have previously been shown to have sex-specific associations with health outcomes and treatment response [14], [17], [18]. While both men and women have historically derived clinically meaningful benefit from obesity medications [14], the higher percentage of body fat in women and the distinct adipose tissue distribution pattern may result in differential treatment response over time compared with men [19]. Consequently, standard BMI cut-offs may not fully capture sex-specific differences in risks of adiposity-related complications.

In this post hoc analysis of the ATTAIN-1 trial, we evaluated the efficacy and safety of orforglipron among individuals with BMI ≥27 to <30 kg/m^2^ and at least one adiposity-related complication, a subgroup increasingly recognized as clinically relevant under the recent approaches to obesity management. We further explored sex-specific associations with efficacy parameters to gain additional insights into treatment-group consistency and to inform obesity management strategies.

## 2 Methods

### 2.1 Study design and participants

This was a post hoc analysis of the ATTAIN-1 trial (ClinicalTrials.gov NCT05869903) [14], a phase 3, multinational, randomized, double-blind, placebo-controlled trial conducted in 137 sites and 9 countries evaluating once-daily oral orforglipron as an adjunct to healthy diet and physical activity for 72 weeks. In the primary trial, eligible adults did not have diabetes and had either obesity (BMI ≥30 kg/m^2^) or overweight (BMI ≥27 kg/m^2^) with at least one adiposity-related complication (hypertension, dyslipidemia, OSA, or cardiovascular disease). The primary endpoint of the ATTAIN-1 trial was the percent change in body weight from baseline to Week 72, assessed by the treatment-regimen estimand in the intention-to-treat population. Detailed study design and results have been published previously [14].

This post hoc analysis of the ATTAIN-1 trial comprised of participants with BMI ≥27 to <30 kg/m^2^ and at least one adiposity-related complication. For the purposes of this analysis, the BMI <30 kg/m^2^ subgroup was operationalized as BMI ≤29.9 kg/m^2^. This resulted in the exclusion of 16 participants with BMI >29.9 and < 30 kg/m^2^ range.

### 2.2 Endpoints and assessments

In this post hoc analysis, efficacy endpoints included change from baseline to Week 72 the percent change in body weight, change in BMI and waist circumference, categorical weight-loss responders (≥5%, ≥10%, and ≥15%), systolic and diastolic blood pressure, fasting lipids (total, low-density lipoprotein [LDL]-, non-HDL-, and HDL-cholesterol and triglycerides), and high-sensitivity C-reactive protein (hs-CRP). Target-based endpoints included reaching HDL-cholesterol targets (≥50 mg/dL in women, ≥40 mg/dL in men), triglycerides <150 mg/dL, a composite of waist-to-height ratio <0.53 with BMI <27 kg/m^2^, BMI <25 kg/m^2^, and composite blood pressure control (systolic <130 mmHg and diastolic <80 mmHg) at Week 72. Efficacy endpoints also included concomitant antihypertensive and lipid-lowering medication status at Week 72 relative to baseline. Patient-reported outcomes comprised change from baseline to Week 72 in the IWQOL-Lite-CT [20] and the SF-36v2 [21]. IWQoL-Lite-CT is a 20-item patient-reported outcome instrument that assesses the impact of body weight on quality of life across Physical, Physical Function, and Psychosocial Self-esteem domains and a Total score; Scores range from 0 to 100, with higher scores indicating better functioning and quality of life [20]; SF-36v2 validated generic patient-reported outcome instrument that assesses health-related quality of life across 8 domains and summarizes physical and mental health using the Physical Component Summary (PCS) and Mental Component Summary (MCS) scores. Scoring of each domain and both summary scores are norm based and presented in the form of T-scores, with a mean of 50 and standard deviation of 10; higher scores indicate better levels of function and/or better health [21]. Safety endpoints were treatment-emergent adverse events (TEAEs), serious adverse events, adverse events leading to treatment discontinuation, deaths, gastrointestinal adverse events, and discontinuation due to nausea, vomiting, or diarrhea. All efficacy endpoints were analyzed for the overall population, as well as for sex-based subgroups.

### 2.3 Statistical analysis

The primary estimand for this post hoc analysis was the efficacy estimand, representing the treatment effect assuming that all randomized patients adhered to the orforglipron or placebo administration as intended and did not initiate prohibited weight-management therapies. Thus, the assessments guided by the efficacy estimand included all data points from randomized participants obtained during the treatment period and up to the earliest date of treatment discontinuation or initiation of prohibited weight management medications. Continuous efficacy endpoints were assessed using a maximum likelihood-based mixed-model for repeated measures (MMRM), including all scheduled postbaseline visits. The MMRM model included the variables region, strata, baseline value of the dependent variable, treatment-by-visit interaction, baseline-by-treatment-by-visit interaction, and strata-by-treatment-by-visit interaction. Lipid and hs-CRP parameters were log-transformed before fitting the model, and baseline was log-transformed before being added as a covariate. Model-based estimates (MBEs), 95% CIs, and treatment differences were back-transformed and presented as mean percent change from baseline and as relative treatment difference to placebo in percent change. Missing values were implicitly handled by the MMRM under the missing-at-random assumption. For binary endpoints derived from continuous variables, missing values of the continuous variable were imputed first under the assumption of missing at random using multiple imputation by treatment group, and the corresponding binary variable was then derived. A logistic regression model was used to examine the treatment difference, including treatment group, region, strata, the continuous baseline value, treatment-by-strata interaction, and treatment-by-baseline interaction. Treatment effects were assessed using risk differences estimated via the delta method [22]. The treatment regimen estimand was evaluated as a supportive analysis; details are provided in the Supplementary methods. All p-values are nominal as they were not tested for multiplicity.

## 3 Results

### 3.1 Participant disposition, baseline demographics, and clinical characteristics

ATTAIN-1 (NCT05869903) was a phase 3, randomized, double-blind, placebo-controlled trial in which adults without diabetes and with obesity or overweight were randomized 3:3:3:4 to once-daily oral orforglipron 5.5, 9, or 17.2 mg or placebo for 72 weeks. This post hoc analysis included the subset of randomized participants with a BMI of 27 or more and below 30 kg/m^2^ and at least one adiposity-related complication (total N = 260; orforglipron 5.5 mg [n = 56], 9 mg [n = 64], 17.2 mg [n = 62], and placebo [n = 78]) (Fig. 1). Overall, 205 (78.8%) participants (orforglipron 5.5 mg [n = 51; 91.1%], 9 mg [n = 51; 79.7%], 17.2 mg [n = 48; 77.4%], or placebo [n = 55; 70.5%]) completed the 72-week treatment period. The most common reasons for treatment discontinuation were adverse events (orforglipron 5.5 mg [n = 1; 1.8%], 9 mg [n = 6; 9.4%], 17.2 mg [n = 6; 9.7%], or placebo [n = 5; 6.4%]) and withdrawal by the participant (orforglipron 5.5 mg [n = 4; 7.1%], 9 mg [n = 6; 9.4%], 17.2 mg [n = 4; 6.5%], or placebo [n = 12; 15.4%]). In total, 225 (86.5%) participants completed the study.

**Fig. 1:**
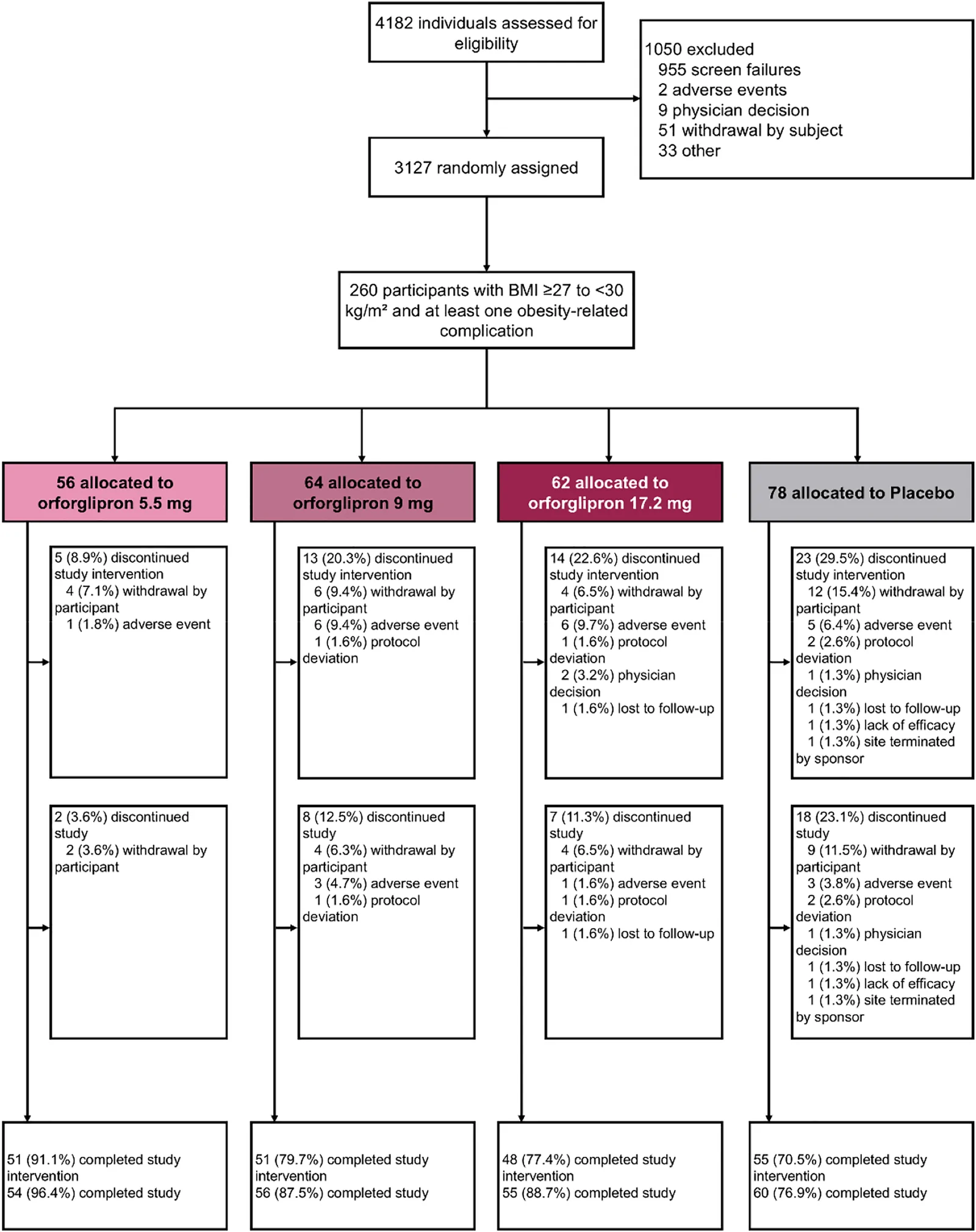
Disposition of ATTAIN-1 Participants with BMI of 27 or more and below 30 kg/m^2^ and Adiposity-Related Complications.

The female participant subgroup included 31 (55.4%), 29 (45.3%), 28 (45.2%), and 36 (46.2%) participants in orforglipron 5.5 mg, 9 mg, 17.2 mg, and placebo groups, respectively. The male participant subgroup included 25 (44.6%), 35 (54.7%), 34 (54.8%), and 42 (53.8%) participants in orforglipron 5.5 mg, 9 mg, 17.2 mg, and placebo groups, respectively.

Study completion is defined as completing both the 72-week treatment period and the safety follow-up period for participants without prediabetes at randomization. For participants with prediabetes at randomization, study completion is defined as completing the 72-week treatment period.

Baseline demographics and clinical characteristics in this post hoc analysis were balanced across treatment groups (Table 1). The mean age (standard deviation [SD]) of the participants was 47.8 (12.4), 51.2 (11.9), 49.9 (10.9), and 52.4 (11.8) years for orforglipron 5.5 mg, 9 mg, 17.2 mg, and placebo, respectively (Table 1). Participants were predominantly Asian (5.5 mg: 80.4%; 9 mg: 76.6%; 17.2 mg: 77.4%; placebo: 78.2%), and the majority of participants were from Japan and China (Table S1). Prediabetes was present in 37.5%, 39.1%, 32.3%, and 50% of participants in the orforglipron 5.5 mg, 9 mg, 17.2 mg, and placebo, respectively. Overall, adiposity-related complication rates were generally balanced across treatment groups. Dyslipidemia (orforglipron 5.5 mg [76.8%], 9 mg [76.6%], or 17.2 mg [75.8%], or placebo [75.6%]) and hypertension (orforglipron 5.5 mg [48.2%], 9 mg [54.7%], or 17.2 mg [54.8%], or placebo [56.4%]) were the most prevalent adiposity-related complications (Table 1).

**Table 1:** Baseline characteristics in ATTAIN-1 Participants with BMI of 27 or more and below 30 kg/m^2^ and Adiposity-Related Complications.

Characteristic	Orforglipron 5.5 mg (N = 56)	Orforglipron 9 mg (N = 64)	Orforglipron 17.2 mg (N = 62)	Placebo (N = 78)
**Age, years**	47.8 (12.4)	51.2 (11.9)	49.9 (10.9)	52.4 (11.8)
**Sex, n (%)**				
Female	31 (55.4)	29 (45.3)	28 (45.2)	36 (46.2)
Male	25 (44.6)	35 (54.7)	34 (54.8)	42 (53.8)
**Race**^a^**, n (%)**				
Asian	45 (80.4)	49 (76.6)	48 (77.4)	61 (78.2)
White	8 (14.3)	14 (21.9)	13 (21.0)	16 (20.5)
Black or African American	1 (1.8)	1 (1.6)	0	1 (1.3)
Multiple	2 (3.6)	0	1 (1.6)	0
**Ethnicity**^a^**, n (%)**				
Hispanic or Latino	6 (10.7)	4 (6.3)	3 (4.8)	5 (6.4)
Not Hispanic or Latino	50 (89.3)	60 (93.8)	59 (95.2)	73 (93.6)
**Body weight, kg**	80.6 (9.0)	79.9 (9.3)	80.2 (8.2)	79.9 (9.6)
**BMI, kg/m^2^**	28.8 (0.8)	28.5 (0.8)	28.4 (0.8)	28.6 (0.8)
**Waist circumference, cm**	97.0 (6.7)	97.6 (6.1)	98.0 (4.9)	97.8 (6.5)
**Blood pressure, mm Hg**				
Systolic	119.6 (13.1)	125.3 (11.7)	119.9 (15.4)	122.0 (11.8)
Diastolic	77.1 (8.4)	82.1 (9.2)	77.7 (10.5)	80.0 (9.6)
**Pulse rate, bpm**	72.9 (9.6)	74.2 (10.6)	71.9 (9.7)	72.2 (10.4)
**Lipid parameters, mg/dL**^a^				
Triglycerides	134.2 (51.5)	140.2 (56.1)	131.1 (54.3)	132.7 (50.8)
Total cholesterol	196.5 (23.0)	200.6 (19.3)	200.4 (20.7)	205.5 (17.4)
LDL-cholesterol	116.6 (33.9)	112.5 (37.8)	118.2 (29.6)	122.6 (25.3)
HDL-cholesterol	47.6 (24.2)	51.5 (24.0)	50.4 (23.0)	51.0 (25.1)
Non-HDL cholesterol	146.8 (28.0)	146.1 (26.5)	147.3 (27.7)	151.7 (23.6)
**Lipid parameters, mmol/L**^a^				
Triglycerides	1.5 (51.5)	1.6 (56.1)	1.5 (54.3)	1.5 (50.8)
Total cholesterol	5.1 (23.0)	5.2 (19.3)	5.2 (20.7)	5.3 (17.4)
LDL-cholesterol	3.0 (33.9)	2.9 (37.8)	3.1 (29.6)	3.2 (25.3)
HDL-cholesterol	1.2 (24.2)	1.3 (24.0)	1.3 (23.0)	1.3 (25.1)
Non-HDL cholesterol	3.8 (28.0)	3.8 (26.5)	3.8 (27.7)	3.9 (23.6)
**Hs-CRP, mg/L**^a^	1.7 (118.3)	1.1 (118.1)	1.2 (154.4)	1.1 (110.9)
**Prediabetes, n (%)**	21 (37.5)	25 (39.1)	20 (32.3)	39 (50.0)
**Comorbidity, n (%)**				
Dyslipidemia	43 (76.8)	49 (76.6)	47 (75.8)	59 (75.6)
Hypertension	27 (48.2)	35 (54.7)	34 (54.8)	44 (56.4)
Gout/hyperuricemia	9 (16.1)	10 (15.6)	10 (16.1)	16 (20.5)
Obstructive sleep apnea	6 (10.7)	4 (6.3)	12 (19.4)	9 (11.5)
MASLD	13 (23.2)	20 (31.3)	14 (22.6)	27 (34.6)

Data are mean (SD) for the given units unless otherwise stated. Race and ethnic groups were reported by the patients.

Abbreviations: BMI = body mass index; CV = coefficient of variation; HbA1c = glycated hemoglobin A1c; HDL = high-density lipoprotein; hs-CRP = high-sensitivity C-reactive protein; LDL = low-density lipoprotein; MASLD = metabolic dysfunction-associated steatotic liver disease; n = number of participants in that subgroup; SD = standard deviation.

a Baseline lipids and hs-CRP are presented as geometric mean (percentage of CV).

At baseline, BMI was similar between males and females. Compared with males, females had lower body weight, waist circumference and diastolic blood pressure; while having higher total cholesterol, LDL-C, HDL-C, and non-HDL-C, and lower triglycerides. Females also had lower rates of prediabetes, gout or hyperuricemia, OSA and MASLD compared to males (Table S2).

### 3.2 Efficacy

Greater observed mean percent changes in body weight were observed in orforglipron 5.5 mg, 9 mg, and 17.2 mg groups compared with placebo. Changes were observed from the first dose escalation at Week 4 (−1.5% to −1.9% with orforglipron [all groups at 0.8 mg at this timepoint] compared to 0.0% with placebo) and maintained throughout intervention. At Week 72, the mean percent changes from baseline in body weight were −7.4% (95% confidence interval [CI]: −9.0, −5.8), −10.3% (95% CI: −12.0, −8.5) and −13.5% (95% CI: −16.0, −10.9) in orforglipron 5.5 mg, 9 mg, and 17.2 mg groups, respectively, compared to −0.3% (95% CI: −1.6, 1.0) with placebo (Fig. 2A). The estimated treatment differences (ETD) were −7.1% (95% CI: −9.2, −5.0), −10.0% (95% CI: −12.2, −7.7), and −13.2% (95% CI: −16.1, −10.2) for the 5.5 mg, 9 mg, and 17.2 mg doses, respectively (all nominal p < 0.001 versus placebo). BMI reductions from baseline were also greater in orforglipron groups versus placebo (all doses nominal p < 0.001 versus placebo at Week 72) (Fig. 2B). Females exhibited numerically greater reductions in body weight across all treatment groups (orforglipron 5.5 mg: −9.7%, 95% CI: −12.2, −7.2; orforglipron 9 mg: −12.5%, 95% CI: −15.7, −9.4; orforglipron 17.2 mg: −18.8%, 95% CI: −23.2, −14.4; all nominal p < 0.001 versus placebo) compared with males (orforglipron 5.5 mg: −4.8%, 95% CI: −6.8, −2.8, nominal p < 0.01 versus placebo; orforglipron 9 mg: −8.2%, 95% CI: −10.0, −6.3, nominal p < 0.001 versus placebo; orforglipron 17.2 mg: −10.0%, 95% CI: −12.8, −7.2, nominal p < 0.001 versus placebo) (Fig. 2C and D). BMI reductions with orforglipron were up to −4.9 kg/m^2^ in female participants, and −2.8 kg/m^2^ in males (Fig. S1A and S1B).

**Fig. 2:**
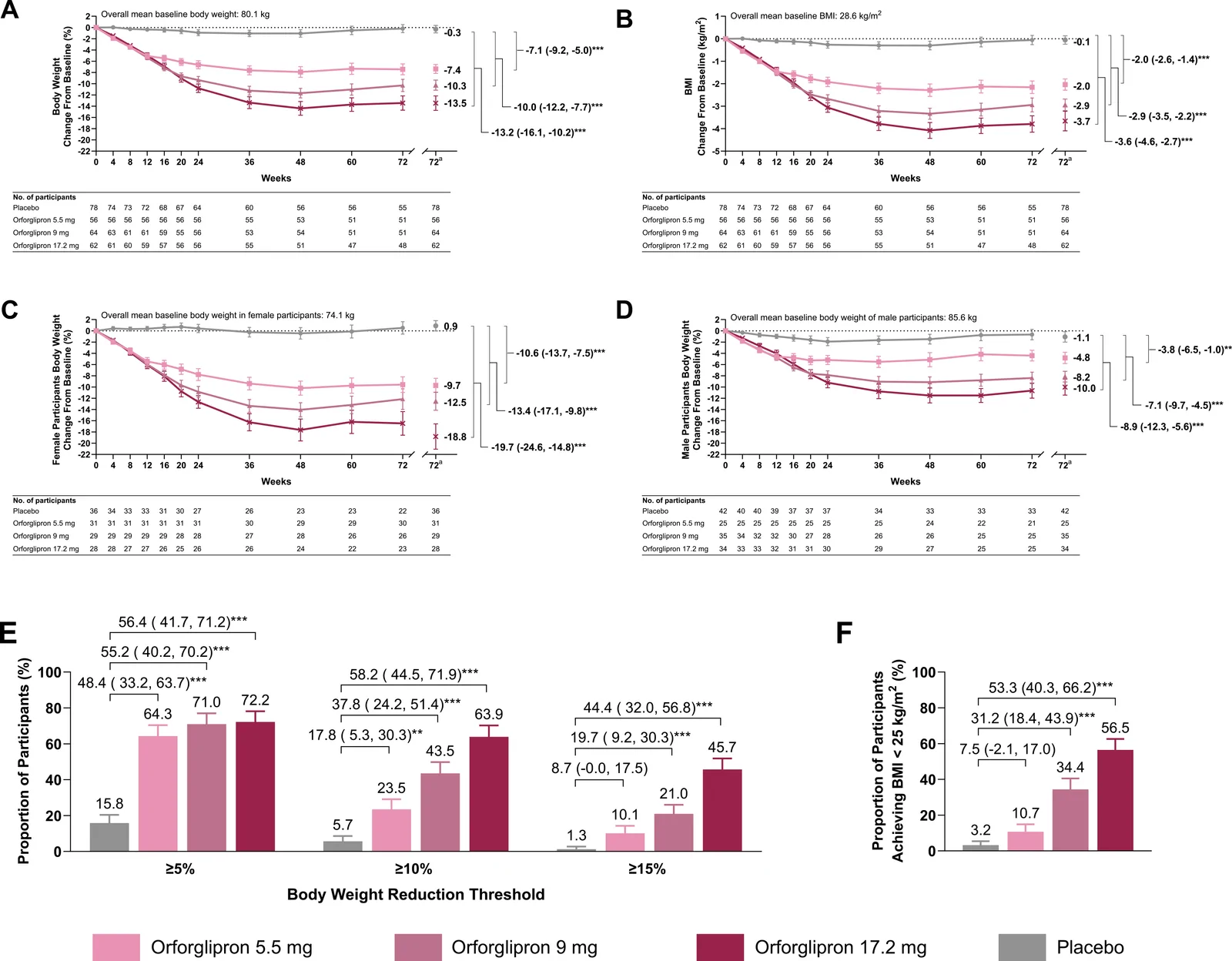
Body Weight, BMI, and Participants Reaching Body Weight Reduction Thresholds in ATTAIN-1 Participants with BMI of 27 or more and Below 30 kg/m^2^ and Adiposity-Related Complications. (A) Over time curve data are observed mean percent change from baseline in body weight for the efficacy estimand; text data at Week 72 is MBE change from baseline for the efficacy estimand with ETD (95% CI) versus placebo. (B) Over time curve data are observed mean change from baseline in BMI for the efficacy estimand; text data at Week 72 is MBE change from baseline for the efficacy estimand with ETD (95% CI) versus placebo. (C and D) Over time curve data are observed mean percent change from baseline in body weight for the efficacy estimand in female participants (C) and male participants (D), respectively; text data at Week 72 is MBE change from baseline for the efficacy estimand with ETD (95% CI) versus placebo. (E) Proportion of participants reaching body weight reduction targets of ≥5%, ≥10%, and ≥15% at Week 72 from logistic regression analysis for the efficacy estimand with RD (95% CI) versus placebo from logistic regression. (F) Proportion of participants reaching BMI of <25 kg/m^2^ at Week 72 from logistic regression analysis for the efficacy estimand with RD (95% CI) versus placebo from logistic regression. Error bars denote SE. All p-values are nominal. *p < 0.05, **p < 0.01, ***p < 0.001 for the respective orforglipron group versus placebo at Week 72. aEfficacy estimand MBE (95% CI) and ETD at Week 72 between orforglipron groups and placebo based on a mixed model for repeated measures analysis. Abbreviations: BMI = body mass index; CI = confidence interval; ETD = estimated treatment difference; MBE = model-based estimate; RD = risk difference; SE = standard error.

More participants reached body weight reduction targets of ≥5%, ≥10%, and ≥15% in orforglipron groups than with placebo, with the numerically greatest response observed in the orforglipron 17.2 mg group (72.2% reaching 5% body weight reduction, 63.9% reaching 10%, and 45.7% reaching 15%, all nominal p < 0.001 versus placebo) compared with placebo (15.8%, 5.7%, 1.3%, respectively) (Fig. 2E). At Week 72, no participant in any treatment group became underweight. Among female participants, 77.9%, 70.2%, and 63.2% reached body weight reduction thresholds of 5%, 10% or 15% in the orforglipron 17.2 mg group, respectively, compared to 11.1%, 2.8%, and 0.2% with placebo, respectively (Fig. S1C; Table S3). Among male participants, 68.1%, 59.3%, and 31.7% reached body weight reduction thresholds of 5%, 10% or 15% in the orforglipron 17.2 mg group, respectively, compared to 20.5%, 7.9%, and 2.2% with placebo, respectively (Fig. S1D; Table S3). Participants achieving a BMI <25 kg/m^2^ at Week 72 were 10.7% in the orforglipron 5.5 mg group (ETD 7.5%, 95% CI: −2.1, 17.0 versus placebo), 34.4% in the orforglipron 9 mg group (ETD 31.2%, 95% CI: 18.4, 43.9, nominal p < 0.001 versus placebo); and 56.5% in the orforglipron 17.2 mg group (ETD 53.3%, 95% CI: 40.3, 66.2, nominal p < 0.001 versus placebo) (Fig. 2F). Female participants achieving a BMI <25 kg/m^2^ were 18.7%, 44.5%, and 64.0% in orforglipron 5.5 mg, 9 mg and 17.2 mg groups, respectively, compared to 2.5% with placebo (Fig. S1E). Male participants achieving a BMI <25 kg/m^2^ were 2.7%, 26.5%, and 50.2% in orforglipron 5.5 mg, 9 mg and 17.2 mg groups, respectively, compared to 3.5% with placebo (Fig. S1F). Similar results were observed with the treatment regimen estimand for overall population (Table S4) and female and male subgroups (Table S5).

Greater changes from baseline to Week 72 in waist circumference were observed in all orforglipron groups (5.5 mg: −6.0 cm, 9 mg: −8.4 cm, 17.2 mg: −10.9 cm) versus placebo (−1.5 cm). Numerically greater reductions from baseline to Week 72 were observed in higher orforglipron dose groups, separating from placebo within the first 4 weeks and continuing through Week 72 (all doses nominal p < 0.001 versus placebo at Week 72) (Fig. 3A). In female participants, change from baseline in waist circumference ranged from −7.5 cm to −15.4 cm in orforglipron groups, and was −1.7 cm with placebo; in male participants it ranged from −3.8 to −6.9 cm in orforglipron groups, and was −1.8 cm with placebo (Fig. S2A and S2B).

**Fig. 3:**
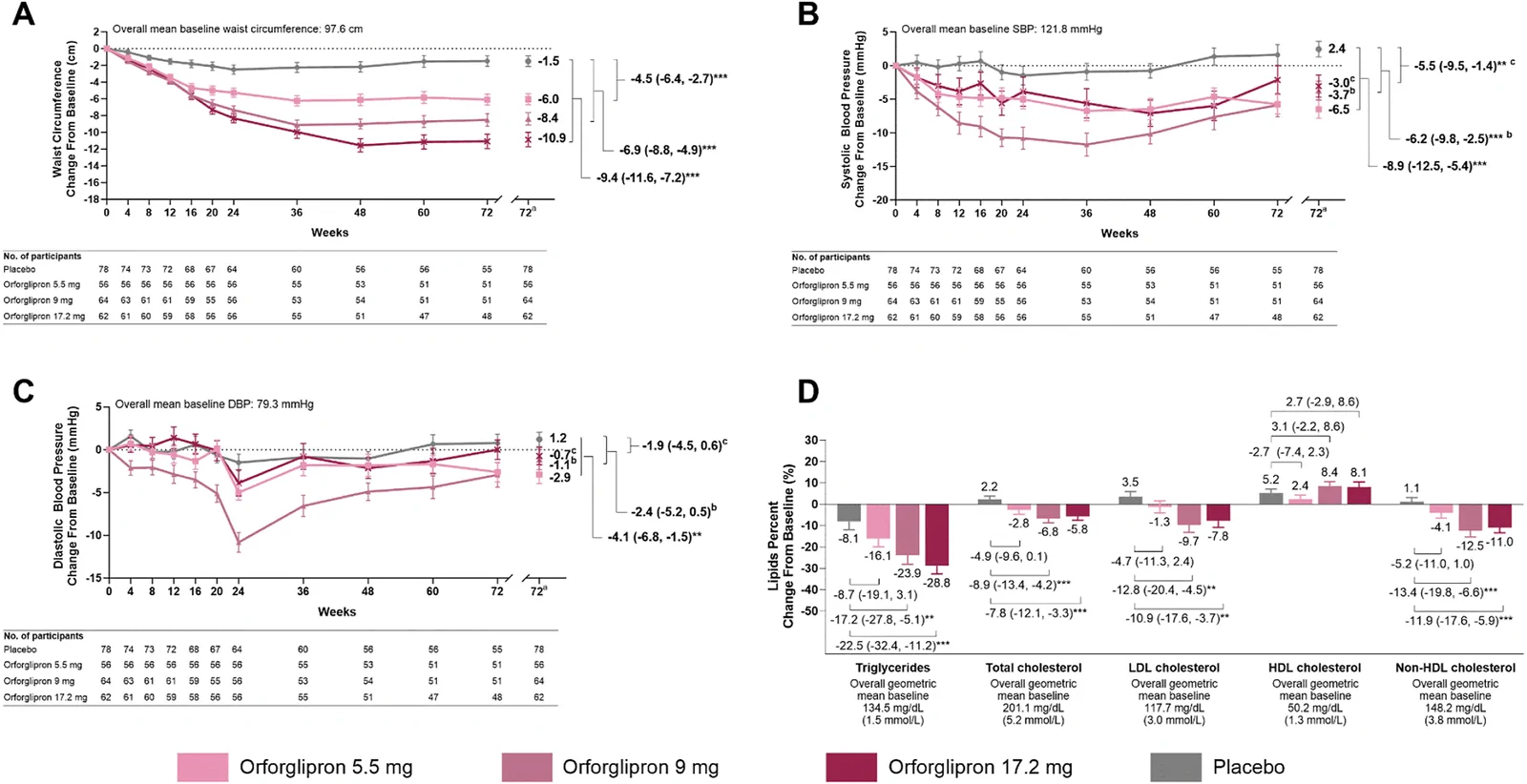
Waist Circumference, Blood Pressure and Lipid parameters in ATTAIN-1 Participants with BMI of 27 or more and below 30 kg/m^2^ and Adiposity-Related Complications. (A) Over time curve data are observed mean change from baseline in waist circumference for the efficacy estimand; text data at Week 72 is MBE change from baseline for the efficacy estimand with ETD (95% CI) versus placebo. (B) Over time curve data are observed mean change from baseline in systolic blood pressure for the efficacy estimand; text data at Week 72 is MBE change from baseline for the efficacy estimand with ETD (95% CI) versus placebo. (C) Over time curve data are observed mean change from baseline in diastolic blood pressure for the efficacy estimand; text data at Week 72 is MBE change from baseline for the efficacy estimand with ETD (95% CI). (D) MBE percent change from baseline in lipid parameters and ETD (95% CI) versus placebo at Week 72 from MMRM for the efficacy estimand. Error bars denote SE. All p-values are nominal as they were not tested for multiplicity. *p < 0.05, **p < 0.01, ***p < 0.001 for the respective orforglipron group versus placebo at Week 72. ^a^Efficacy estimand MBE (95% CI) and ETD at Week 72 between orforglipron groups and placebo based on a mixed model for repeated measures analysis. ^b^Denotes orforglipron 9 mg group where datapoints overlap. ^c^Denotes orforglipron 17.2 mg group where datapoints overlap. Abbreviations: CI = confidence interval; ETD = estimated treatment difference; HDL = high-density lipoprotein; LDL = low-density lipoprotein; MBE = model-based estimate; SE = standard error.

Reduction from baseline in systolic blood pressure (SBP) was greater in orforglipron groups (all doses) than in placebo groups (all doses nominal p < 0.01 versus placebo at Week 72, Fig. 3B), change from baseline in diastolic blood pressure (DBP) was comparable across all treatment groups at Week 72 (Fig. 3C). Changes in blood pressure in orforglipron groups were generally comparable between females and males (Fig. S2). Greater percent reduction from baseline in lipid parameters were observed across orforglipron groups compared to placebo, with lower triglycerides, total cholesterol, non-high-density lipoprotein (non-HDL cholesterol), and low-density lipoprotein (LDL-cholesterol) at Week 72. HDL cholesterol increased from baseline; no difference was observed across treatment groups (Fig. 3D). Changes in lipid parameters were generally comparable between females and males (Fig. S3). Reduction from baseline in hs-CRP ranged from −22.4% to −31.5% in orforglipron groups, and was −18.2% with placebo, there was no difference across treatment groups; change from baseline was up to −52.4% in female participants, and up to −23.8% in male participants (Fig. S4). Similar results were observed with the treatment regimen estimand for overall population (Table S4) and female and male subgroups (Table S5).

#### 3.2.1 Cardiometabolic responder analyses

At Week 72, participants treated with orforglipron demonstrated meaningful improvements in cardiometabolic parameters (Table 2). Participants achieving a waist-to-height ratio <0.53 and BMI <27 kg/m^2^ were 28.7%, 39.5%, and 57.1% in the orforglipron 5.5 mg, 9 mg, and 17.2 mg groups, respectively, compared with 6.4% in the placebo group (all nominal p < 0.01 versus placebo) (Table 2); responder rates in orforglipron groups were numerically higher among female (39.1%–77.8%) than male participants (32.9%–50.1%) (Table S6). Participants achieving blood pressure control (SBP <130 and DBP <80 mmHg) were 61.3%, 55.8%, 58.6%, in the orforglipron 5.5 mg, 9 mg, 17.2 mg groups, respectively, compared with 43.7% in the placebo group (nominal p < 0.05 for orforglipron 5.5 mg versus placebo) (Table 2); responder rates in orforglipron groups were numerically higher among female (53.2%–70.8%) than male participants (48.0%–57.1%) (Table S6). There were no meaningful differences among treatment groups in proportions of participants achieving triglyceride <150 mg/dL responders and HDL target responders (≥50 mg/dL in females/≥40 mg/dL in males) (Table 2). Across all treatment groups, triglyceride responder rates were numerically higher in female (70.1%–84.7%) than male participants (61.8%–74.8%); and sex-adjusted HDL responder rates were numerically lower in female (67.3%–77.4%) than male participants (77.2%–90.6%) (Table S6). Similar results were observed for cardiometabolic parameters with the treatment regimen estimand for overall population (Table S7) and for female and male subgroups (Table S8).

**Table 2:** Cardiometabolic Responders Subgroups Analyses in ATTAIN-1 Participants with BMI of 27 or more and below 30 kg/m^2^ and Adiposity-Related Complications.

Parameter	Orforglipron 5.5 mg (N = 56)	Orforglipron 9 mg (N = 64)	Orforglipron 17.2 mg (N = 62)	Placebo (N = 78)
**HDL Target responders (≥50 mg/dL Female/≥40 mg/dL Male)**				
Group mean (%) (SE)	77.2 (4.4)	83.3 (4.4)	78.3 (5.2)	76.1 (5.0)
RD vs. Placebo (95% CI), p-Value	1.0 (−10.9, 13.0), p = 0.865	7.2 (−5.4, 19.7), p = 0.260	2.2 (−12.0, 16.4), p = 0.762	–
**Triglycerides <150 mg/dL Responders**				
Group mean (%) (SE)	75.8 (5.7)	69.5 (5.8)	76.8 (5.4)	65.6 (5.4)
RD vs. Placebo (95% CI), p-Value	10.1 (−5.3, 25.5), p = 0.197	3.9 (−11.6, 19.3), p = 0.622	11.2 (−3.7, 26.1), p = 0.139	–
**Waist-to-height ratio <0.53 & BMI <27 kg/m^2^ responders**				
Group mean (%) (SE)	28.7 (5.6)	39.5 (6.1)	57.1 (6.0)	6.4 (3.1)
RD vs. Placebo (95% CI), p-Value	22.3 (9.5, 35.0), p < 0.001	33.1 (19.7, 46.5), p < 0.001	50.7 (37.4, 64.0), p < 0.001	–
**BMI <27 kg/m^2^ responders**				
Group mean (%) (SE)	51.3 (6.7)	74.3 (5.5)	73.8 (5.6)	15.4 (4.2)
RD vs. Placebo (95% CI), p-Value	35.9 (20.2, 51.6), p < 0.001	58.9 (45.3, 72.6), p < 0.001	58.4 (44.9, 72.0), p < 0.001	–
**Systolic blood pressure <130 mm Hg and diastolic blood pressure <80 mm Hg responders**				
Group mean (%) (SE)	61.3 (5.9)	55.8 (6.5)	58.6 (6.4)	43.7 (5.6)
RD vs. Placebo (95% CI), p-Value	17.6 (2.0, 33.2), p = 0.028	12.1 (−3.6, 27.8), p = 0.129	14.8 (−1.5, 31.1), p = 0.074	–

Statistical inference for endpoint used Rubin's Rule to combine analyses of imputed datasets. G-computation was used to estimate the unconditional average treatment effect. Group mean, risk difference, CI, and p-value for endpoint measures are from logistic regression model using imputed data. Logistic regression model: Variable = Baseline + Treatment + Geographic Region + Strata (defined by joint levels of sex [female, male] and prediabetes status [yes, no]) + Treatment*Strata + Treatment*Baseline. All p-values are nominal as they were not tested for multiplicity. Abbreviations: BMI = body mass index; CI = confidence interval; HDL = high-density lipoprotein; RD = risk difference; SE = standard error.

#### 3.2.2 Patient-reported outcomes

Patient-reported outcomes from baseline to Week 72 improved across all treatment groups, and greater improvements were observed in orforglipron versus placebo groups. All IWQOL-Lite-CT domains improved in orforglipron 9 mg and 17.2 mg groups, with the greatest improvements observed for the psychosocial composite score (change from baseline [95% CI]): orforglipron 5.5 mg: 7.0 (3.8, 10.2); 9 mg: 11.2 (8.6, 13.9); 17.2 mg: 14.1 (11.5, 16.7); and placebo: 4.9 (1.7, 8.1) (Fig. 4A). Across the SF-36v2 components, improvements versus placebo were observed in the orforglipron 9 mg group for the physical component score (ETD 1.6, 95% CI: 0.0, 3.3, nominal p < 0.05) and general health (ETD 1.8, 95% CI: 0.0, 3.5, nominal p < 0.05); and in the orforglipron 5.5 mg group for physical functioning (ETD 1.4, 95% CI: 0.1, 2.7, nominal p < 0.05). No differences across orforglipron treatment groups versus placebo were observed for the SF-36v2 mental component score, role-physical, bodily pain, vitality, social functioning, role-emotional, and mental health (Fig. 4B). Female participants in orforglipron groups experienced numerically greater improvements with orforglipron than males in all IWQOL-Lite-CT components, and in SF-36-v2 physical component score, physical functioning and general health (Fig. S5). Similar results and inferences were observed with the treatment regimen estimand for overall population (Table S9) and for female and male subgroups (Table S10).

**Fig. 4:**
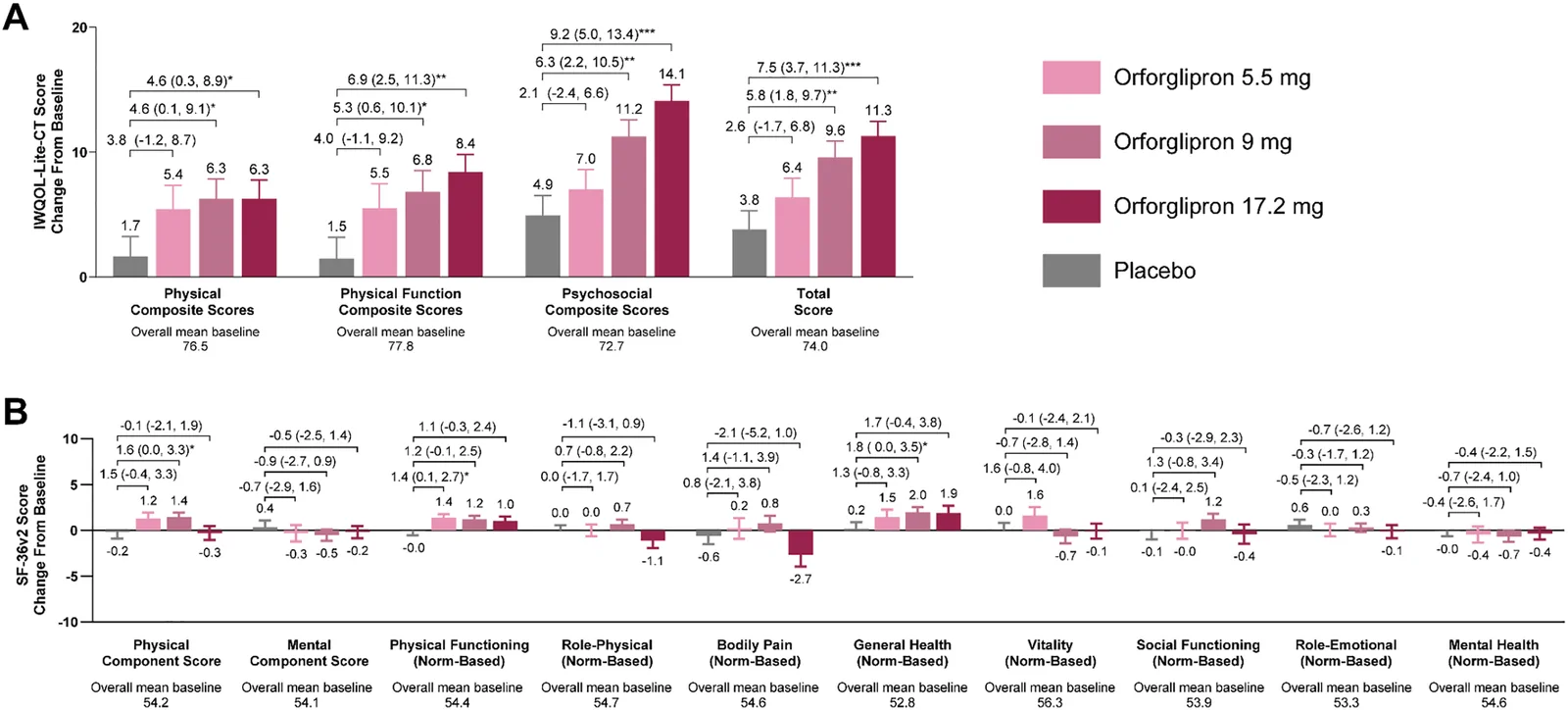
Patient-Reported Outcomes in ATTAIN-1 Participants with BMI of 27 or more and below 30 kg/m^2^ and Adiposity-Related Complications. (A) MBE change from baseline in IWQOL-Lite-CT scores and ETD (95% CI) versus placebo at Week 72 from MMRM for the efficacy estimand. (B) MBE change from baseline in SF-36v2 scores and ETD (95% CI) versus placebo at Week 72 from MMRM for the efficacy estimand. Error bars denote SE. All p-values are nominal as they were not tested for multiplicity. *p < 0.05, **p < 0.01, ***p < 0.001 for the respective orforglipron group versus placebo at Week 72. Abbreviations: CI = confidence interval; ETD = estimated treatment difference; IWQOL-Lite-CT = Impact of weight on quality of life-lite clinical trials; MBE = model-based estimate; MMRM = mixed model for repeated measures; SE = standard error; SF-36v2 = Short-form-36 version 2.

#### 3.2.3 Concomitant therapy

At Week 72, the use of concomitant antihypertensive therapy was similar across treatment groups during the trial, with proportions of 28.6%, 45.3%, and 29.0% in the orforglipron 5.5 mg, 9 mg, and 17.2 mg groups, respectively, compared with 33.3% in the placebo arm. Decreasing (3.6%, 4.7%, 3.2%) or stopping therapy due to no longer needing it (1.8%, 3.1%, 4.8%) occurred only in the orforglipron groups (5.5 mg, 9 mg, and 17.2 mg, respectively); and more participants experienced increases in therapy in the placebo group (4 participants in placebo group versus 1 participants in orforglipron 17.2 mg group). A similar pattern was observed for concomitant lipid-lowering therapy, with 25.0%, 37.5%, and 25.8% of participants in the orforglipron 5.5 mg, 9 mg, and 17.2 mg groups, respectively, compared with 21.8% in the placebo group. In contrast, decreased therapy was reported in 4.8% of participants at 17.2 mg dose, and stopping therapy due to no longer needing it in 1.6% of participants in both the orforglipron 9 mg and 17.2 mg groups, respectively, compared with none in the placebo arm. (Table S11). Similar trends were observed across female and male participants (Table S12).

### 3.3 Safety

Overall, the proportions of participants reporting at least one TEAE were similar across treatment groups, with 76.8%, 85.9%, 79.0%, and 82.1% of participants in the orforglipron 5.5 mg, 9 mg, 17.2 mg, and placebo groups, respectively (Table 3). Serious AEs were reported by 1.6%–7.8% of participants treated with orforglipron, and in 9.0% of those in the placebo group (Table 3). Discontinuation due to AEs were 1.8%–9.7% across orforglipron groups, and 6.4% in placebo group. There were no reports of AEs leading to death.

**Table 3:** Adverse Events in ATTAIN-1 Participants with BMI of 27 or more and below 30 kg/m^2^ and Adiposity-Related Complications.

Adverse event	Orforglipron 5.5 mg (N = 56)	Orforglipron 9 mg (N = 64)	Orforglipron 17.2 mg (N = 62)	Placebo (N = 78)
**Any adverse event emerging during treatment period**	43 (76.8)	55 (85.9)	49 (79.0)	64 (82.1)
**Serious adverse event**	3 (5.4)	5 (7.8)	1 (1.6)	7 (9.0)
**Death**	0	0	0	0
**Event leading to discontinuation of orforglipron or placebo**				
Any adverse event	1 (1.8)	6 (9.4)	6 (9.7)	5 (6.4)
Gastrointestinal disorder	1 (1.8)	2 (3.1)	4 (6.5)	0
**Adverse events of special interest and other safety topics**				
Hepatic disorders^a^	1 (1.8)	0	0	0
Cancer	1 (1.8)	2 (3.1)	0	3 (3.8)
Adjudication-confirmed pancreatitis	0	0	0	0
Hypotension, orthostatic hypotension, and syncope^a^	0	0	0	0
Adjudication-confirmed MACE	0	0	0	0
Any cardiac disorder^b^	1 (1.8)	0	0	0
Gastrointestinal event^a^	2 (3.6)	1 (1.6)	1 (1.6)	0
Gallbladder and biliary tract disorders^a^	1 (1.8)	1 (1.6)	0	0
Acute renal events^a^	0	0	0	0
Major depressive disorder or suicidal ideation or behavior	0	0	0	0
Hypersensitivity^a^	0	0	0	1 (1.3)
Dysesthesia	0	0	1 (1.6)	0
**Other adverse events emerging during treatment period**				
Cholelithiasis	0	1 (1.6)	0	0
Acute cholecystitis	0	0	0	0
Chronic cholecystitis	1 (1.8)	1 (1.6)	0	0

Data are n (%).

Abbreviations: AE = adverse event; GI = gastrointestinal; MACE = major adverse cardiovascular events; TEAE = treatment-emergent adverse event.

a Events that were classified as severe or serious adverse events.

b Cardiac disorders included events that were classified as severe or serious arrhythmias and cardiac conduction disorders.

Gastrointestinal AEs were the most frequently reported TEAEs, and were more frequent with orforglipron than with placebo, with 60.7%, 64.1%, 50.0%, and 33.3% of participants in the orforglipron 5.5 mg, 9 mg, 17.2 mg, and placebo groups, respectively (Table 3). The most common gastrointestinal AEs were nausea, vomiting, constipation, and diarrhea, and were predominantly mild-to-moderate in severity. (Table S13). The incidence of nausea, vomiting, and diarrhea over time is presented in Fig. S6. AEs leading to treatment discontinuation were reported among 1.8%–9.7% of the participants in the orforglipron groups and in 6.4% of those in the placebo group (Table S14). Treatment discontinuation due to GI AEs occurred in 1.8%, 3.1% and 6.5% of participants treated with orforglipron 5.5 mg, 9 mg, and 17.2 mg, respectively (Table S14); among which 1.8%, 1.6% and 4.8% were due to nausea/vomiting/diarrhea, respectively. Change from baseline at Week 72 in pulse rate, SBP, and DBP for the safety data set are presented in Table S15.

## 4 Discussion

In this post hoc analysis of ATTAIN-1 participants with BMI ≥27 to <30 kg/m^2^ and at least one adiposity-related complication, orforglipron was associated with clinically meaningful reductions in body weight, BMI, and waist circumference and improvements in several cardiometabolic parameters and patient-reported quality of life outcomes relative to placebo through Week 72. The safety profile was characterized by predominantly mild-to-moderate gastrointestinal AEs. These improvements were broadly consistent with those reported for the overall ATTAIN-1 population [14], despite this subgroup starting closer to a healthy-weight threshold.

The present findings support the relevance of obesity medication treatment in people with BMI ≥27 to <30 kg/m^2^ and adiposity-related complications, as orforglipron was associated with clinically meaningful weight and cardiometabolic improvements in this lower-BMI, complication-bearing population that were consistent with efficacy findings in the broader population studied in ATTAIN-1 [14]. At the highest orforglipron dose of 17.2 mg, more than half of participants achieved a BMI <25 kg/m^2^, crossing below the conventional BMI threshold for overweight. This high proportion of participants achieving a BMI <25 kg/m^2^ likely reflects, in part, that participants in this subgroup initiated treatment at a lower BMI and therefore closer to the BMI 25 kg/m^2^ threshold than the overall ATTAIN-1 population. Given the predominance of Asian participants, achieving BMI <25 kg/m^2^ should not be interpreted as indicating absence of obesity or cardiometabolic risk. Rather, this finding illustrates the substantial magnitude of weight reduction achieved in this lower-BMI population. For instance, population-based data indicate that the risk of developing prediabetes is 24% higher in people with overweight or obesity compared to those with healthy weight [23], therefore supporting the potential benefits of intervening early in overweight and obesity progression.

The waist-circumference reductions observed in this subgroup may carry similar early-intervention value, as well as the higher rates of participants achieving the composite endpoint of waist-to-height ratio <0.53 and BMI <27 kg/m^2^ in orforglipron groups compared to placebo. Central abdominal fat accumulation is a stronger determinant of cardiometabolic disease risk than BMI alone, including in people whose BMI falls below the conventional threshold for obesity [24]. Current guidance recommends routine waist-circumference measurement as a marker of this risk in anyone with a BMI <35 kg/m^2^, a description that applies directly to this subgroup [9]. Moreover, clinically meaningful reductions in waist circumference and cardiometabolic risk factors were observed despite participants having BMI at the lower end of the pharmacotherapy indication spectrum. Therefore, reducing waist circumference at this earlier stage may offer an opportunity to limit future cardiovascular risk before atherosclerotic and other vascular changes become established. Whether the weight and waist-circumference reductions observed in this subgroup would similarly translate into reduced long-term cardiovascular risk was not assessed in this analysis and would require dedicated cardiovascular-outcomes studies.

Baseline differences across treatment groups are worth noting when interpreting these findings. Although demographics were generally balanced, prediabetes prevalence was numerically higher in the placebo group (50.0%) than in the orforglipron groups (32.3%–39.1%), reflecting the study design. Despite having a lower BMI by design, participants had significant adiposity-related complications, highlighting the substantial health burden associated with excess adiposity in this subgroup. Concomitant antihypertensive and lipid-lowering medication use also varied across groups (28.6%–45.3% and 25.0%–37.5% of participants, respectively). Given the modest size of each group (n = 56–78), such imbalances likely reflect chance rather than a systematic effect of randomization, but they should be considered alongside between-group differences in blood pressure and lipid endpoints. The subgroup was also markedly enriched for Asian participants (76.6%–80.4%) and correspondingly underrepresented for White and Hispanic/Latino participants relative to the overall ATTAIN-1 population (28.6% Asian; 37.6% Hispanic/Latino) [14], consistent with epidemiological evidence that Asian populations tend to develop adiposity-related complications at a lower BMI than people of European or African ancestry [25], which may lower the threshold at which they meet criteria such as those used in this subgroup.

An exploratory finding of note was the apparent difference in weight-loss magnitude by sex at the highest dose, with women showing a numerically greater percent reduction in body weight than men (−18.8% versus −10.0%). Consistently, further sex-based subgroup analyses showed that females also tended to experience numerically greater changes in BMI, waist circumference, and hs-CRP, along with numerically greater improvements in IWQOL compared to males, while both subgroups had comparable concomitant medication use. The improvements in lipid parameters observed in the placebo group could be attributed to concomitant lipid-lowering medication used during the trial, as well as healthy lifestyle modifications that were recommended in the protocol and other factors. Sex-based variation in response to incretin-based therapies has been described previously [26], and these observations are directionally consistent with that pattern. There is a growing body of evidence suggesting that females often achieve greater weight loss with GLP-1 RAs and related incretin therapies compared with males. In a systematic review and meta-analysis of 41 articles representing 64 randomized clinical trials, it was observed that GLP-1 RAs effects were substantially greater among women than men [27]. Several mechanisms may contribute to the observed sex-related differences, including potential synergistic interactions between GLP-1 and estrogen, as well as differences in pharmacokinetics and drug exposure resulting from the lower average body weight of women [28], [29], [30]. These findings suggest that the greater mean percent body weight reduction observed in females than males in our analysis may not be solely attributable to the small sample size. However, with 260 participants divided across four groups and two sexes, these comparisons were not powered for formal inference and should be interpreted as hypothesis-generating; they also cannot be generalized for the population.

These findings complement observations from the overall ATTAIN-1 trial [14], in which participants with a BMI of 27–30 kg/m^2^ and an adiposity-related complication were among those most likely to achieve a BMI <25 kg/m^2^ or a lower-risk waist-to-height ratio with orforglipron treatment [14]. The present analysis provides a more detailed characterization of efficacy, and patient-reported outcome in this subgroup along-with sex-specific differences. The consistency of relative treatment effects between this subgroup and the overall trial population supports the interpretation that orforglipron's benefits are not confined to participants with higher BMI. The observed sex-related differences in treatment response suggest that sex may be an important determinant of therapy outcomes.

Adults with overweight and adiposity-related complications may be overlooked or underdiagnosed in clinical practice [2]. These patients may be perceived as having a milder or lower-priority weight concern because their BMI falls below the conventional threshold for obesity, and pharmacologic treatment is frequently deferred until complications become more advanced and/or the patient reaches higher BMI. This perception is increasingly at odds with a contemporary understanding of the disease. The Lancet Commission on clinical obesity argues that the presence of adiposity-related organ dysfunction, rather than BMI category, should determine whether obesity constitutes a serious, chronic illness, and recommends that individuals meeting this threshold receive the same timely, evidence-based care afforded to other chronic, potentially life-threatening diseases [2]. By this framework, adults with overweight and established adiposity-related complication meet the definition of clinical obesity and may already warrant the same clinical seriousness as those with a higher BMI, even though the overweight label itself often leads to less urgent management. The European Association for the Study of Obesity (EASO) staging framework [9], the AACE complication-centric treatment algorithm [10], and Canadian pharmacotherapy guidance [11] converge on this same principle: treatment decisions should be guided by the presence and severity of adiposity-related complications rather than BMI thresholds alone. Therefore, the current analysis provides valuable insight into these contemporary frameworks for obesity management, with dedicated efficacy and safety data for a population that is often deprioritized in clinical practice and underrepresented in obesity trials, despite already carrying the burden of adiposity-related complications.

Taken together, this post hoc analysis suggests that orforglipron was associated with consistent weight and cardiometabolic benefits in adults with BMI ≥27 to <30 kg/m^2^and an adiposity-related complication. These data may help healthcare professionals to inform patients with BMI ≥27 to <30 kg/m^2^ and adiposity-related complications regarding potential benefits associated with orforglipron. Confirmation in larger and more demographically diverse populations is required to confirm the current findings.

## 5 Limitations

Several limitations warrant consideration. This was a post hoc analysis that was not powered for hypothesis testing, and results should be considered hypothesis-generating rather than confirmatory. The subgroup was small (n = 260 total, 56–78 per group), which limits the precision of effect estimates, particularly for lower-frequency endpoints such as HDL-cholesterol and triglyceride target responders, where confidence intervals were wide. The population was predominantly Asian, with limited representation of White, Black, and Hispanic/Latino participants, which may limit generalizability to other populations. The numerically lower rate of gastrointestinal adverse events observed in this subgroup relative to the overall obesity population should not be interpreted as evidence of a more favorable tolerability profile in participants with BMI ≥27 to <30 kg/m^2^ specifically, given the small sample size and absence of formal statistical comparison. Finally, statistical inference was based on model-based estimates and 95% CIs without multiplicity adjustment, consistent with the exploratory nature of the analysis.

## 6 Conclusion

In this post hoc analysis of ATTAIN-1 participants with BMI ≥27 to <30 kg/m^2^ and at least one adiposity-related complication, orforglipron was associated with clinically meaningful weight reduction and improvements in cardiometabolic risk factors. These findings support the utility of orforglipron for treating individuals at the lower end of the BMI range for which obesity pharmacotherapy is indicated.

**Key clinical takeaways**

• Orforglipron was associated with weight loss and cardiometabolic improvement in adults with BMI of ≥27 to <30 kg/m^2^and ≥1 adiposity-related complication that were comparable to those in adults with overweight or obesity from ATTAIN-1 trial.
• More than half of participants at the highest dose achieved BMI <25 kg/m^2^, with a consistent gastrointestinal-predominant safety profile.
• These post hoc analyses support the use of orforglipron for treating excess adiposity in people with BMI of ≥27 to <30 kg/m^2^and with adiposity-related complication but require confirmation in larger, more demographically diverse populations.

## CRediT author statement

All authors have read and agreed to the published version of the manuscript.

**Jaime P Almandoz**: Investigation; Writing – original draft; Writing – review & editing.

**Alexander Miras**: Investigation; Writing – original draft; Writing – review & editing.

**Matthias Blüher**: Investigation; Writing – original draft; Writing – review & editing.

**Pedro Orozco Del Pino**: Conceptualization; Design; Investigation; Data acquisition; Formal analysis; Writing – original draft; Writing – review & editing.

**Donna Mojdami**: Investigation; Formal analysis; Writing – original draft; Writing – review & editing.

**Rachel L Batterham**: Investigation; Formal analysis; Writing – original draft; Writing – review & editing.

**Kevin Trice**: Conceptualization; Design; Investigation; Data acquisition; Formal analysis; Writing – original draft; Writing – review & editing.

**Benjamin Goebel:** Conceptualization; Design; Investigation; Data acquisition; Formal analysis; Writing – original draft; Writing – review & editing.

**Fatih Tangi**: Conceptualization; Design; Investigation; Data acquisition; Formal analysis; Writing – original draft; Writing – review & editing.

## Ethics review

The trial was conducted in accordance with local regulations, the principles of the Declaration of Helsinki, and the International Council for Harmonisation Good Clinical Practice guidelines. The protocol was approved by the institutional review board or independent ethics committee at each participating site, and all participants provided written informed consent. The full study protocol has been published with the ATTAIN-1 primary publication [14]. ClinicalTrials.gov NCT05869903.

## Disclosures

Jaime P Almandoz reported receiving personal fees for consulting and advisory activities from AbbVie, Amgen, Boehringer Ingelheim, Eli Lilly and Company, Haleon, Kailera, Metsera/Pfizer, Novo Nordisk, Rivus Pharmaceuticals, Rhythm Pharmaceuticals, and Wave Life Sciences.

Alexander Miras has received research funding from Anabio, Boehringer Ingelheim, the European Union, Fractyl, Gila, Jon Moulton Charitable Foundation, Medical Research Council, National Institute for Health and Care Research, Eli Lilly, Novo Nordisk, Herbalife and Randox. He has received honoraria for lectures and presentations from Algorithm, AstraZeneca, Boehringer Ingelheim, Currax Pharmaceuticals, Eli Lilly, Ethicon, GI Dynamics, Medtronic, Novo Nordisk, and Screen Health. He is a shareholder in Kiso and the Beyond BMI clinic, which provides clinical obesity care.

Matthias Blüher received honoraria as a consultant and speaker from Abbott, Amgen, AstraZeneca, Bayer, Boehringer Ingelheim, Daiichi-Sankyo, Lilly, Novo Nordisk, Pfizer, and Sanofi, and received payment from Boehringer Ingelheim for his participation on a Data Safety Monitoring Board.

Pedro Orozco Del Pino, Donna Mojdami, Rachel Batterham, Kevin Trice, Benjamin Goebel, and Fatih Tangi are employees and shareholders of Eli Lilly and Company.

## Declaration of artificial intelligence (AI) and AI-assisted technologies

AI was not used during the development of this manuscript.

## Source of funding

This study was funded by 10.13039/100004312Eli Lilly and Company.

## Conflict of interest

**Jaime P Almandoz** reported receiving personal fees for consulting and advisory activities from AbbVie, Amgen, Boehringer Ingelheim, Eli Lilly and Company, Haleon, Kailera, Metsera/Pfizer, Novo Nordisk, Rivus Pharmaceuticals, Rhythm Pharmaceuticals, and Wave Life Sciences.

**Alexander Dimitri Miras** has received research funding from Anabio, Boehringer Ingelheim, the European Union, Fractyl, Gila, Jon Moulton Charitable Foundation, Medical Research Council, National Institute for Health and Care Research, Eli Lilly, Novo Nordisk, Herbalife and Randox. He has received honoraria for lectures and presentations from Algorithm, AstraZeneca, Boehringer Ingelheim, Currax Pharmaceuticals, Eli Lilly, Ethicon, GI Dynamics, Medtronic, Novo Nordisk, and Screen Health. He is a shareholder in Kiso and the Beyond BMI clinic, which provides clinical obesity care.

**Matthias Blüher** received honoraria as a consultant and speaker from Abbott, Amgen, AstraZeneca, Bayer, Boehringer Ingelheim, Daiichi-Sankyo, Lilly, Novo Nordisk, Pfizer, and Sanofi, and received payment from Boehringer Ingelheim for his participation on a Data Safety Monitoring Board.

**Pedro Orozco Del Pino, Donna Mojdami, Rachel L Batterham, Kevin Trice, Benjamin Goebel**, and **Fatih Tangi** are employees and shareholders of Eli Lilly and Company.
