# Supplementary material for: Efficacy and safety of orforglipron in adults with body mass index of 27 to less than 30 kg/m2 and adiposity-related complications: A post hoc analysis of the ATTAIN-1 trial
Source: Obes Pillars. 2026 Oct 14;20:100346. doi: 10.1016/j.obpill.2026.100346 (PMC13697276; doi:10.1016/j.obpill.2026.100346)
Supplement: Supplementary file 1 [file mmc1.pdf]

Supplementary Appendix

Efficacy and safety of orforglipron in adults with body mass index of 27 to less than 30 kg/m^2 and adiposity-related complications: a post hoc analysis of the ATTAIN-1 trial

Jaime P Almandoz MD MBA FTOS[1], Alexander Dimitri Miras MD PhD[2], Matthias Blüher MD PhD[3], Pedro Orozco Del Pino PhD[4], Donna Mojdami MD FRCPC[4], Rachel L Batterham MD PhD[4], Kevin Trice MD MBA[4], Benjamin Goebel MD PhD[4], Fatih Tangi MD PhD[4]

Affiliations:
[1]Department of Internal Medicine, Division of Endocrinology, University of Texas Southwestern Medical Center, Dallas, TX, USA

[2]School of Medicine, Faculty of Life & Health Sciences, Ulster University, UK

[3]Helmholtz Institute for Metabolic, Obesity and Vascular Research (HI-MAG), Helmholtz Center Munich, Leipzig University and University of Leipzig Medical Center, Leipzig, Germany; University of Leipzig Medical Center, Leipzig, Germany.

[4]Eli Lilly and Company, Indianapolis, IN, USA

Corresponding author: Fatih Tangi, MD, PhD

Executive Medical Director – Cardiometabolic Health Business Unit, Orforglipron.

Eli Lilly and Company

tangi_fatih@lilly.com

Table of Contents

Additional Statistical analysis methods 5

Treatment regimen estimand........ 5

List of Supplementary Figures and Tables 6

Supplementary Figure 1: Sex-based Subgroup Analyses of Body Weight, BMI, and Participants Reaching Body Weight Reduction Thresholds in ATTAIN-1 Participants with BMI of 27 or more and below 30 kg/m2 and Adiposity-Related Complications 9

Supplementary Figure 2: Sex-based Subgroup Analyses of Waist Circumference, and Blood Pressure in ATTAIN-1 Participants with BMI of 27 or more and below 30 kg/m^2 and Adiposity-Related Complications 10

Supplementary Figure 3: Sex-based Subgroup Analyses of Lipid parameters in ATTAIN-1 Participants with BMI of 27 or more and below 30 kg/m^2 and Adiposity-Related Complications 12

Supplementary Figure 4: Hs-CRP in ATTAIN-1 Participants with BMI of 27 or more and below 30 kg/m^2 and Adiposity-Related Complications 14

Supplementary Figure 5: Sex-based Subgroup Analyses of Patient-Reported Outcomes in ATTAIN-1 Participants with BMI of 27 or more and below 30 kg/m^2 and Adiposity-Related Complications 15

Supplementary Figure 6: Incidence of Nausea, Vomiting, Diarrhea, and Constipation Over Time in ATTAIN-1 Participants with BMI of 27 or more and below 30 kg/m^2 and Adiposity-Related Complications 16

Supplementary Table 1: Country or Region of Origin of ATTAIN-1 Participants with BMI of 27 or more and below 30 kg/m^2 and Adiposity-Related Complications 17

Supplementary Table 2: Sex-based Subgroups Baseline Characteristics in ATTAIN-1 Participants with BMI of 27 or more and below 30 kg/m^2 and Adiposity-Related Complications 18

Supplementary Table 3: Sex-based Subgroups Analyses of Efficacy Endpoints for the Efficacy Estimand in ATTAIN-1 Participants with BMI of 27 or more and below 30 kg/m^2 and Adiposity-Related Complications 20

Supplementary Table 4: Efficacy Endpoints for the Treatment Regimen Estimand in ATTAIN-1 Participants with BMI of 27 or more and below 30 kg/m^2 and Adiposity-Related Complications 24

Supplementary Table 5: Sex-based Subgroups Analyses of Efficacy Endpoints for the Treatment Regimen Estimand in ATTAIN-1 Participants with BMI of 27 or more and below 30 kg/m^2 and Adiposity-Related Complications 27

Supplementary Table 6. Sex-based Subgroups Analyses of Cardiometabolic Responders Subgroups Analyses in ATTAIN-1 Participants with BMI of 27 or more and below 30 kg/m^2 and Adiposity-Related Complications for the Efficacy Estimand 31

Supplementary Table 7. Cardiometabolic Responders Subgroups Analyses in ATTAIN-1 Participants with BMI of 27 or more and below 30 kg/m^2 and Adiposity-Related Complications for the Treatment Regimen Estimand 33

Supplementary Table 8. Sex-based Subgroups Analyses of Cardiometabolic Responders Subgroups Analyses in ATTAIN-1 Participants with BMI of 27 or more and below 30 kg/m^2 and Adiposity-Related Complications for the Treatment Regimen Estimand... 34

Supplementary Table 9: Patient Reported Outcomes at Week 72 for the Treatment Regimen Estimand in ATTAIN-1 Participants with BMI of 27 or more and below 30 kg/m^2 and Adiposity-Related Complications 36

Supplementary Table 10: Sex-based Subgroups Analyses of Patient Reported Outcomes at Week 72 for the Treatment Regimen Estimand in ATTAIN-1 Participants with BMI of 27 or more and below 30 kg/m^2 and Adiposity-Related Complications 38

Supplementary Table 11: Concomitant Antihypertensive Therapy and Lipid-Lowering Therapy in ATTAIN-1 Participants with BMI of 27 or more and below 30 kg/m^2 and Adiposity-Related Complications at Week 72 41

Supplementary Table 12: Sex-based Subgroups Analyses of Concomitant Antihypertensive Therapy and Lipid-Lowering Therapy in ATTAIN-1 Participants with BMI of 27 or more and below 30 kg/m^2 and Adiposity-Related Complications at Week 72 42

Supplementary Table 13: Summary of Adverse Events by Maximum Severity in ATTAIN-1 Participants with BMI of 27 or more and below 30 kg/m^2 and Adiposity-Related Complications 43

Supplementary Table 14: Adverse Events Leading to Study Intervention Discontinuation in ATTAIN-1 Participants with BMI of 27 or more and below 30 kg/m^2 and Adiposity-Related Complications . 45

Supplementary Table 15: Vital Signs at Week 72 for the Safety Data Set in ATTAIN-1 Participants with BMI of 27 or more and below 30 kg/m^2 and Adiposity-Related Complications 46

Supplementary References 47

Additional Statistical analysis methods

Treatment regimen estimand

The assessments guided by the treatment regimen estimand were conducted using the treatment regimen estimand data points set, including all data points from randomized participants obtained during the treatment period, regardless of study treatment discontinuation or initiation of prohibited weight management treatments. For the assessment of continuous efficacy measures guided by the treatment regimen estimand, data for participants with missing values at Week 72 were imputed, and then an ANCOVA model was used with a variance estimator that is robust to model misspecification and heteroscedasticity. The ANCOVA model included variables of treatment group (each dose of orforglipron and placebo), region, strata (defined by joint levels of sex and prediabetes status [Yes or No]), baseline value of the dependent variable, treatment-by-strata interaction, and treatment-by-baseline interaction. For binary efficacy measures derived from a continuous variable and guided by the treatment regimen estimand, the missing value in the underlying continuous variable were imputed first and then the corresponding binary variable was derived. For the assessment of binary efficacy measures the same logistic regression model as for the efficacy estimand was used. Occurrences of missing data at Week 72 were imputed in a fashion consistent with what the values would likely have been had they been collected. Missing data due to permanent discontinuation of study treatment were imputed by treatment group using retrieved dropouts. Additional details regarding missing-data imputation, and statistical methods are provided in the main publication (Wharton *et al*. 2025).

List of Supplementary Figures and Tables

Supplementary Figure 1: Sex-based Subgroup Analyses of Body Weight, BMI, and Participants Reaching Body Weight Reduction Thresholds in ATTAIN-1 Participants with BMI of 27 or more and below 30 kg/m^2 and Adiposity-Related Complications

Supplementary Figure 2: Sex-based Subgroup Analyses of Waist Circumference, and Blood Pressure in ATTAIN-1 Participants with BMI of 27 or more and below 30 kg/m^2 and Adiposity-Related Complications

Supplementary Figure 3: Sex-based Subgroup Analyses of Lipid parameters in ATTAIN-1 Participants with BMI of 27 or more and below 30 kg/m^2 and Adiposity-Related Complications

Supplementary Figure 4: Hs-CRP in ATTAIN-1 Participants with BMI of 27 or more and below 30 kg/m^2 and Adiposity-Related Complications

Supplementary Figure 5: Sex-based Subgroup Analyses of Patient-Reported Outcomes in ATTAIN-1 Participants with BMI of 27 or more and below 30 kg/m^2 and Adiposity-Related Complications

Supplementary Figure 6: Incidence of Nausea, Vomiting, Diarrhea, and Constipation Over Time in ATTAIN-1 Participants with BMI of 27 or more and below 30 kg/m^2 and Adiposity-Related Complications

Supplementary Table 1: Country or Region of Origin of ATTAIN-1 Participants with BMI of 27 or more and below 30 kg/m^2 and Adiposity-Related Complications

Supplementary Table 2: Sex-based Subgroups Baseline Characteristics in ATTAIN-1 Participants with BMI of 27 or more and below 30 kg/m^2 and Adiposity-Related Complications

Supplementary Table 3: Sex-based Subgroups Analyses of Efficacy Endpoints for the Efficacy Estimand in ATTAIN-1 Participants with BMI of 27 or more and below 30 kg/m^2 and Adiposity-Related Complications

Supplementary Table 4: Efficacy Endpoints for the Treatment Regimen Estimand in ATTAIN-1 Participants with BMI of 27 or more and below 30 kg/m^2 and Adiposity-Related Complications

Supplementary Table 5: Sex-based Subgroups Analyses of Efficacy Endpoints for the Treatment Regimen Estimand in ATTAIN-1 Participants with BMI of 27 or more and below 30 kg/m^2 and Adiposity-Related Complications

Supplementary Table 6. Sex-based Subgroups Analyses of Cardiometabolic Responders Subgroups Analyses in ATTAIN-1 Participants with BMI of 27 or more and below 30 kg/m^2 and Adiposity-Related Complications for the Efficacy Estimand

Supplementary Table 7. Cardiometabolic Responders Subgroups Analyses in ATTAIN-1 Participants with BMI of 27 or more and below 30 kg/m^2 and Adiposity-Related Complications for the Treatment Regimen Estimand

Supplementary Table 8. Sex-based Subgroups Analyses of Cardiometabolic Responders Subgroups Analyses in ATTAIN-1 Participants with BMI of 27 or more and below 30 kg/m^2 and Adiposity-Related Complications for the Treatment Regimen Estimand

Supplementary Table 9: Patient Reported Outcomes at Week 72 for the Treatment Regimen Estimand in ATTAIN-1 Participants with BMI of 27 or more and below 30 kg/m2 and Adiposity-Related Complications

Supplementary Table 10: Sex-based Subgroups Analyses of Patient Reported Outcomes at Week 72 for the Treatment Regimen Estimand in ATTAIN-1 Participants with BMI of 27 or more and below 30 kg/m^2 and Adiposity-Related Complications

Supplementary Table 11: Concomitant Antihypertensive Therapy and Lipid-Lowering Therapy in ATTAIN-1 Participants with BMI of 27 or more and below 30 kg/m^2 and Adiposity-Related Complications at Week 72

Supplementary Table 12: Sex-based Subgroups Analyses of Concomitant Antihypertensive Therapy and Lipid-Lowering Therapy in ATTAIN-1 Participants with BMI of 27 or more and below 30 kg/m^2 and Adiposity-Related Complications at Week 72

Supplementary Table 13: Summary of Adverse Events by Maximum Severity in ATTAIN-1 Participants with BMI of 27 or more and below 30 kg/m^2 and Adiposity-Related Complications

Supplementary Table 14: Adverse Events Leading to Study Intervention Discontinuation in ATTAIN-1 Participants with BMI of 27 or more and below 30 kg/m^2 and Adiposity-Related Complications

Supplementary Table 15: Vital Signs at Week 72 for the Safety Data Set in ATTAIN-1 Participants with BMI of 27 or more and below 30 kg/m^2 and Adiposity-Related Complications

Supplementary Figure 1: Sex-based Subgroup Analyses of Body Weight, BMI, and Participants Reaching Body Weight Reduction Thresholds in ATTAIN-1 Participants with BMI of 27 or more and below 30 kg/m2 and Adiposity-Related Complications

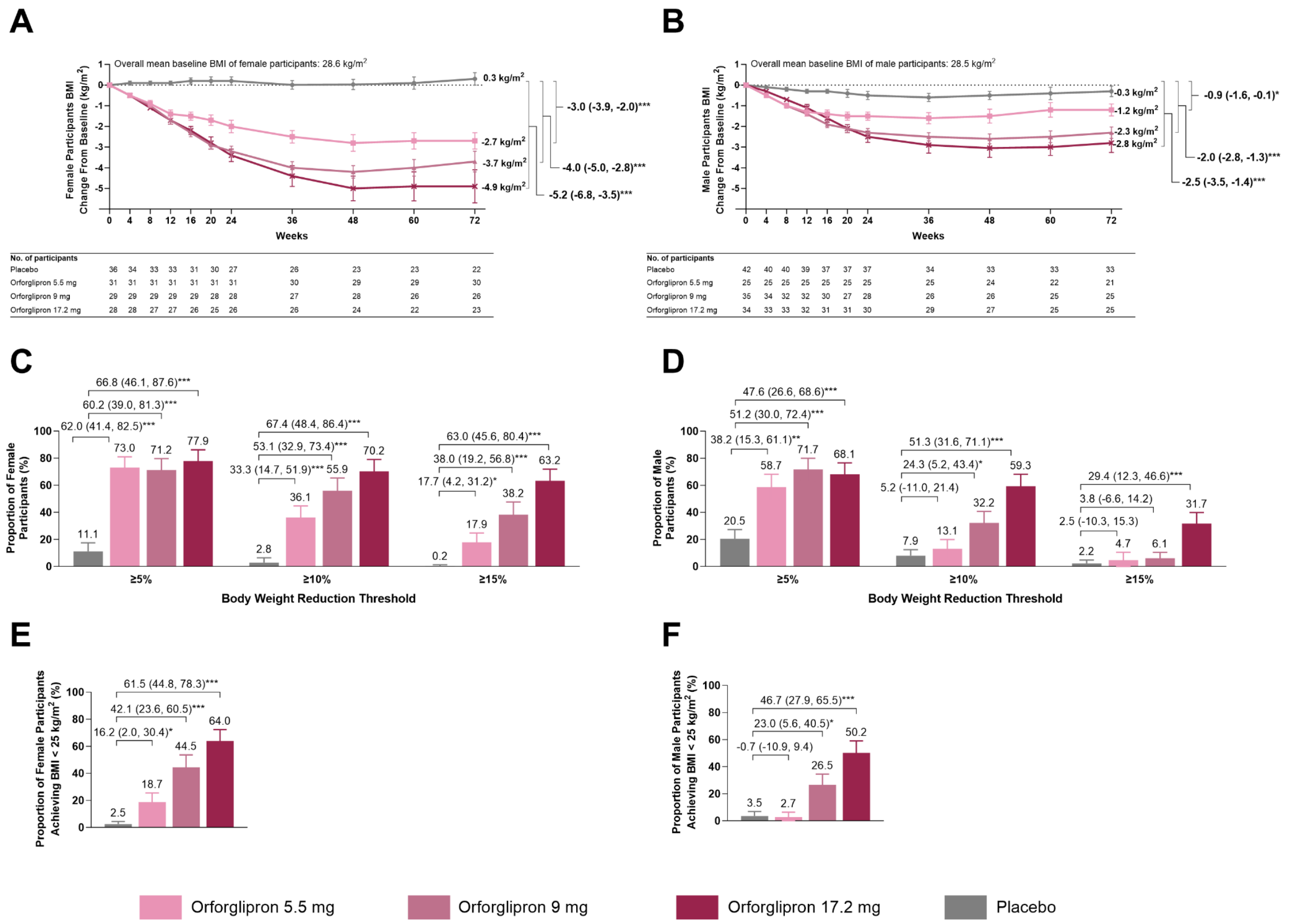

(A and B) Over time curve data are MBE mean change from baseline in BMI among female (A) or male (B) participants for the efficacy estimand with ETD (95% CI) versus placebo at Week 72 based on a mixed model for repeated measures analysis. (C and D) Proportion of female (C) or male (D) participants reaching body weight reduction targets of ≥5%, ≥10%, and ≥15% at Week 72 with RD (95% CI) from logistic regression analysis for the efficacy estimand. (E and F) Proportion of female (E) or male (F) participants reaching BMI of <25 kg/m^2 at Week 72 with RD (95% CI) from logistic regression analysis for the efficacy estimand. Error bars denote SE. All p-values are nominal as they were not tested for multiplicity. *p<0.05, **p<0.01, ***p<0.001 for the respective orforglipron arm versus placebo at Week 72.

Abbreviations: BMI = body mass index; CI = confidence interval; ETD = estimated treatment difference; MBE = model-based estimate; RD = risk difference; SE = standard error

Supplementary Figure 2: Sex-based Subgroup Analyses of Waist Circumference, and Blood Pressure in ATTAIN-1 Participants with BMI of 27 or more and below 30 kg/m^2 and Adiposity-Related Complications

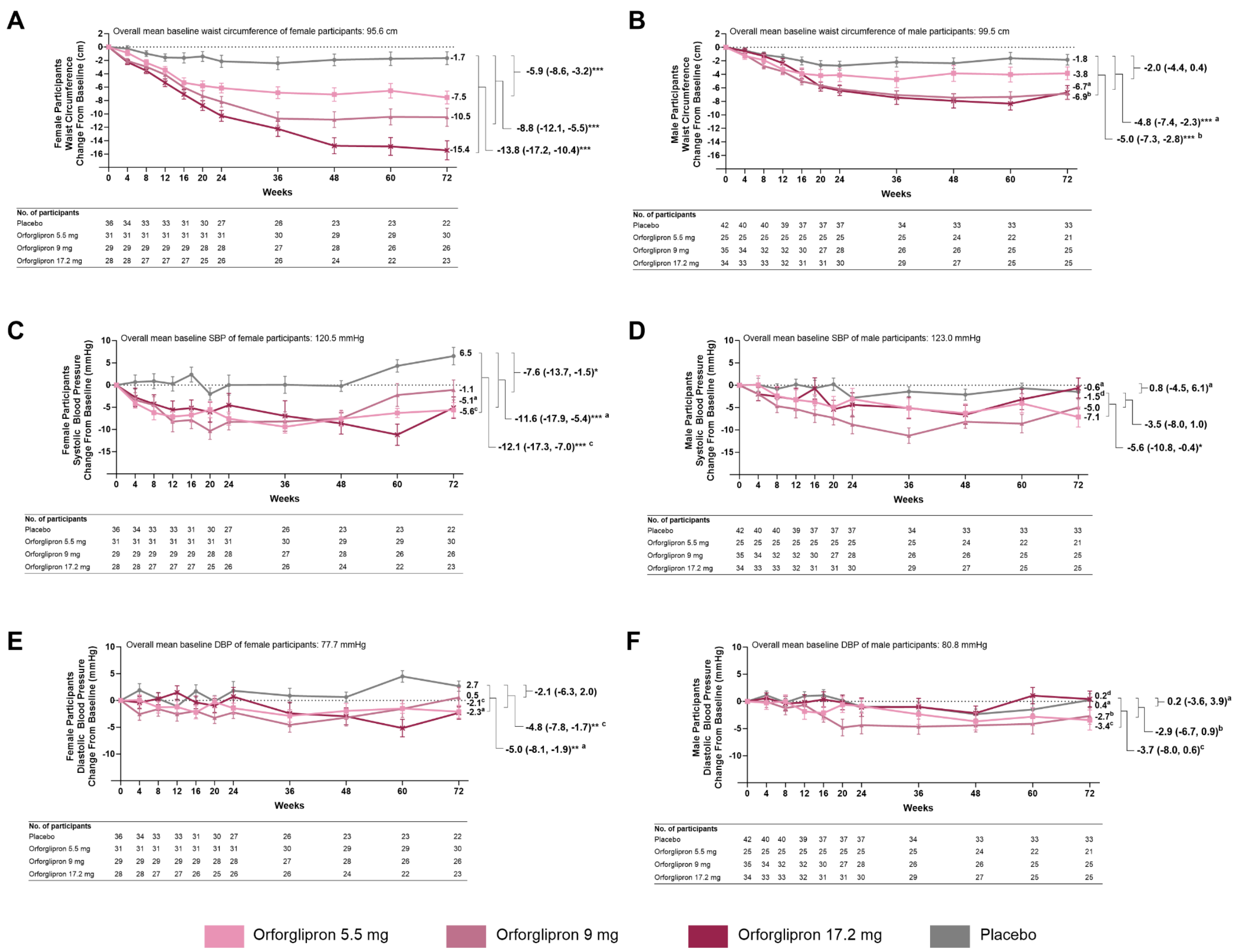

(A and B) Over time curve data are MBE mean change from baseline in waist circumference among female (A) or male (B) participants for the efficacy estimand with ETD (95% CI) versus placebo at Week 72 based on a mixed model for repeated measures analysis. (C and D) Over time curve data are MBE mean change from baseline in systolic blood pressure among female (C) or male (D) participants for the efficacy estimand with ETD (95% CI) versus placebo at Week 72 based on a mixed model for repeated measures analysis. (E and F) Over time curve data are MBE mean change from baseline in diastolic blood pressure among female (E) or male (F) participants for the efficacy estimand with ETD (95% CI) versus placebo at Week 72 based on a mixed model for repeated measures analysis. Error bars denote SE. All p-values are nominal as they were not tested for multiplicity. *p<0.05, **p<0.01, ***p<0.001 for the respective orforglipron arm versus placebo at Week 72.

[a]Denotes orforglipron 17.2 mg arm where datapoints overlap

[b]Denotes orforglipron 9 mg arm where datapoints overlap

[c]Denotes orforglipron 5.5 mg arm where datapoints overlap

[d]Denotes placebo arm where datapoints overlap

Abbreviations: ANOVA = analysis of variance; CI = confidence interval; ETD = estimated treatment difference; MBE = model-based estimate; SE = standard error

Supplementary Figure 3: Sex-based Subgroup Analyses of Lipid parameters in ATTAIN-1 Participants with BMI of 27 or more and below 30 kg/m^2 and Adiposity-Related Complications

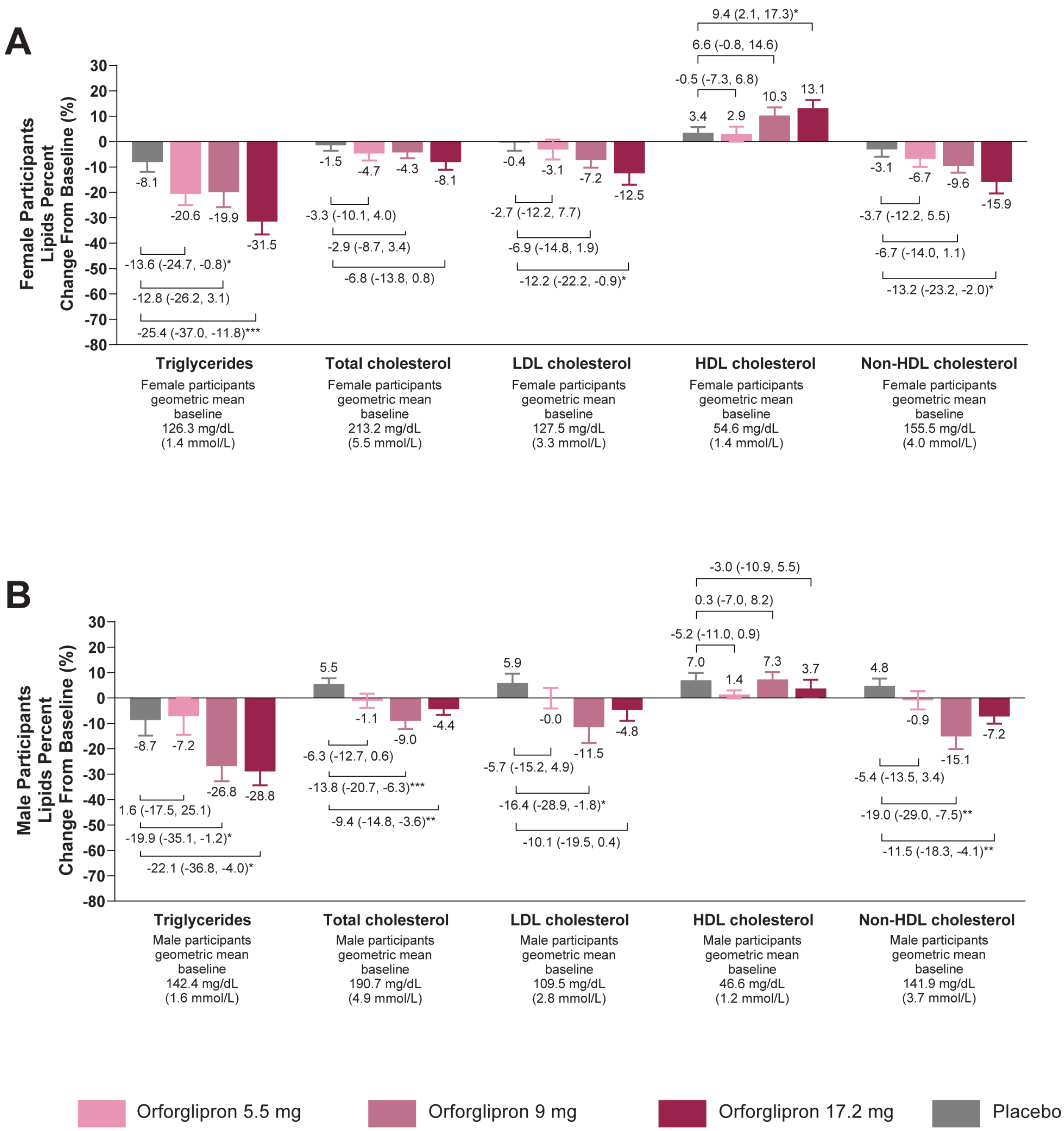

MBE percent change from baseline in lipid parameters among female (A) or male (B) participants from MMRM and ETD (95% CI) versus placebo at Week 72 from ANOVA for the efficacy estimand. Error bars denote SE. All p-values are nominal as they were not tested for multiplicity. *p<0.05, **p <0.01, ***p<0.001 for the respective orforglipron arm versus placebo at Week 72.

Abbreviations: ANOVA = analysis of variance; CI = confidence interval; ETD = estimated treatment difference; HDL = high-density lipoprotein; LDL = low-density lipoprotein; MBE = model-based estimate; MMRM = mixed model for repeated measures; SE = standard error.

Supplementary Figure 4: Hs-CRP in ATTAIN-1 Participants with BMI of 27 or more and below 30 kg/m^2 and Adiposity-Related Complications

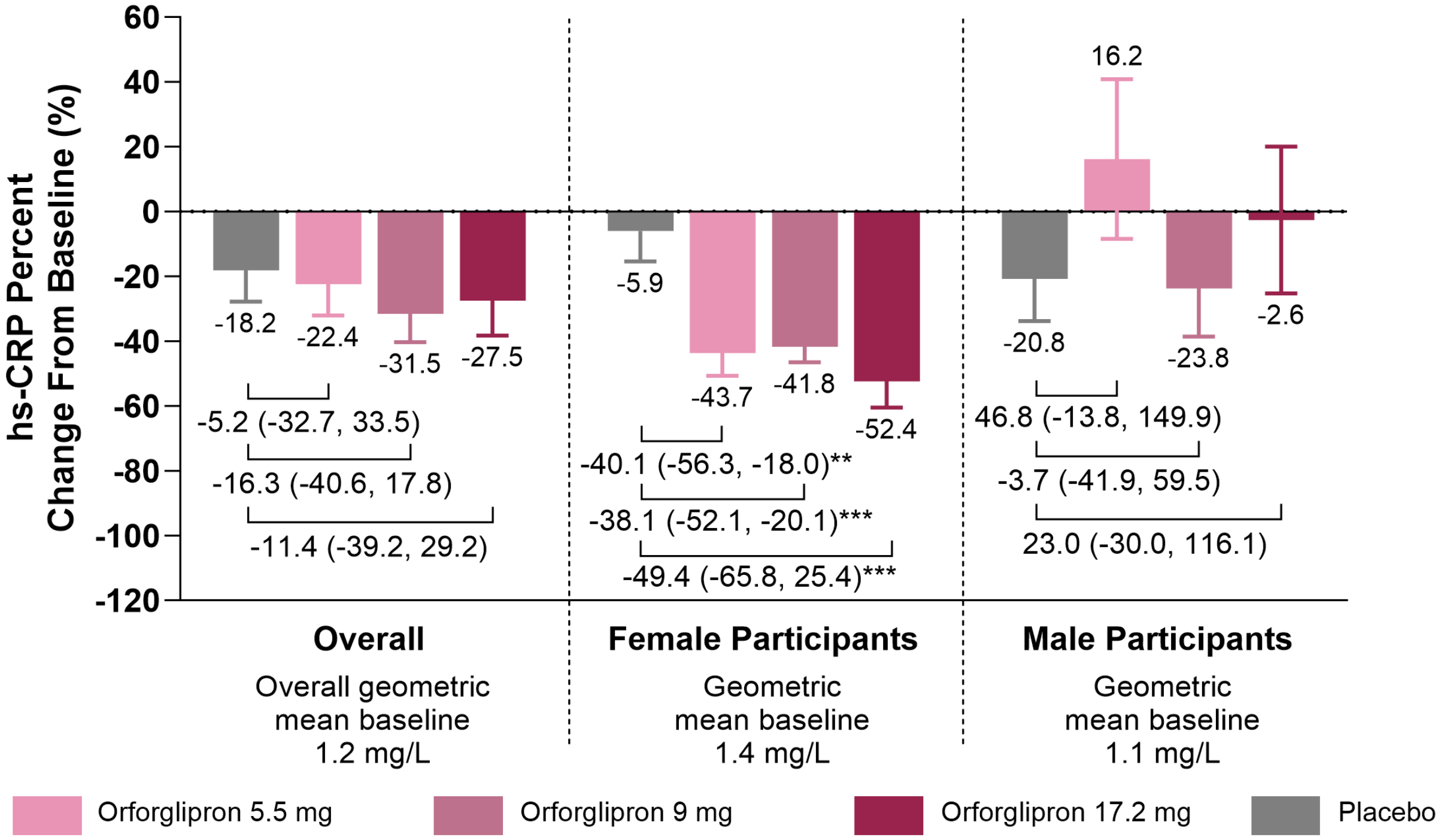

MBE percent change from baseline in hs-CRP from MMRM and ETD (95% CI) versus placebo at Week 72 from ANOVA for the efficacy estimand. Error bars denote SE. All p-values are nominal as they were not tested for multiplicity. **p <0.01, ***p<0.001 for the respective orforglipron arm versus placebo at Week 72.

Abbreviations: ANOVA = analysis of variance; CI = confidence interval; ETD = estimated treatment difference; hs-CRP = high-sensitivity C-reactive protein; MBE = model-based estimate; SE = standard error

Supplementary Figure 5: Sex-based Subgroup Analyses of Patient-Reported Outcomes in ATTAIN-1 Participants with BMI of 27 or more and below 30 kg/m^2 and Adiposity-Related Complications

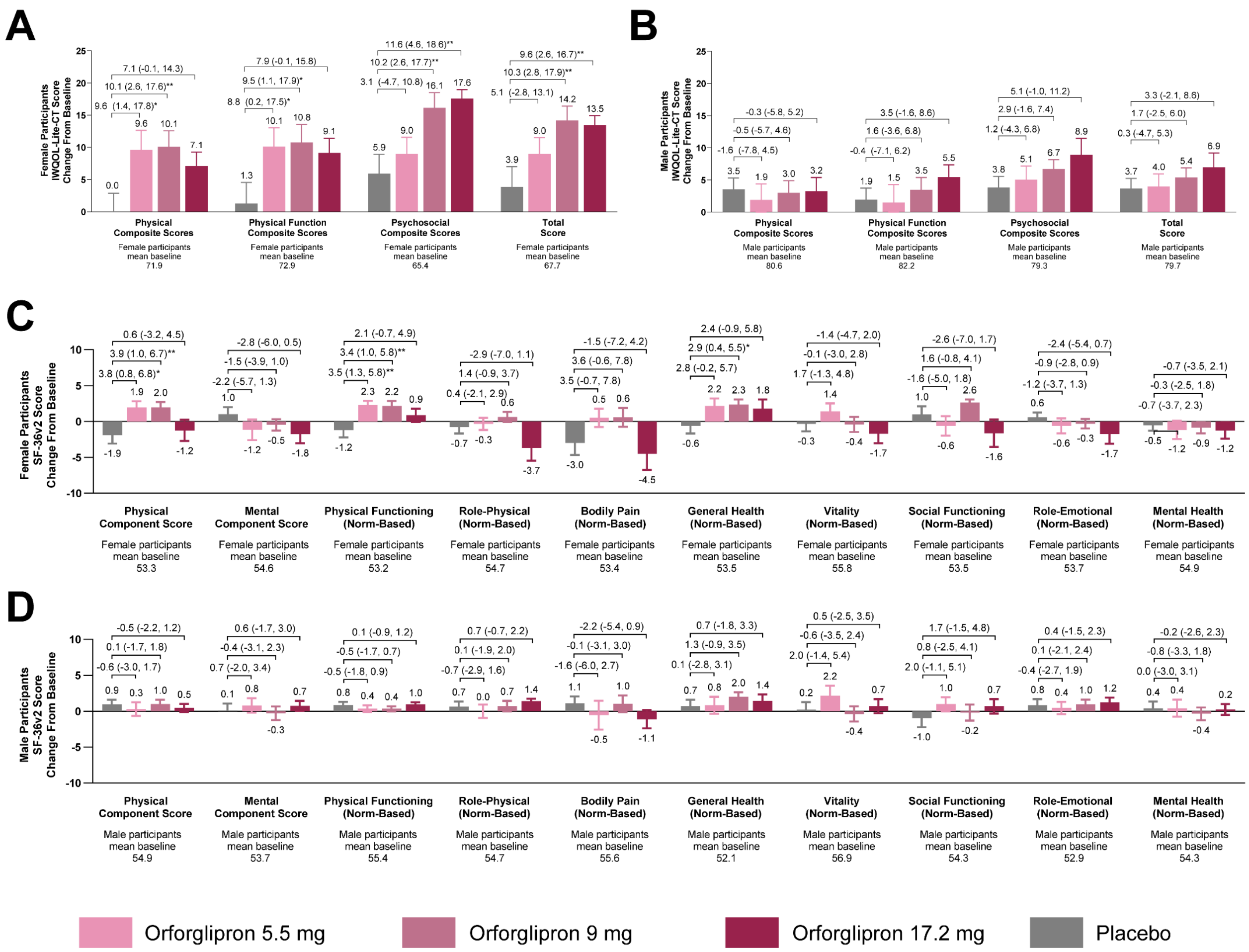

(A and B) MBE percent change from baseline in IWQOL-Lite-CT scores among female (A) and male (B) participants from MMRM and ETD (95% CI) versus placebo at Week 72 from ANOVA for the efficacy estimand. (C and D) MBE percent change from baseline in SF-36v2 scores among female (C) and male (D) participants from MMRM and ETD (95% CI) versus placebo at Week 72 from ANOVA for the efficacy estimand. Error bars denote SE. All p-values are nominal as they were not tested for multiplicity. *p<0.05, ** p <0.01, ***p<0.001 for the respective orforglipron arm versus placebo at Week 72.

Abbreviations: ANOVA = analysis of variance; CI = confidence interval; ETD = estimated treatment difference; IWQOL-Lite-CT = Impact of weight on quality of life-lite clinical trials; MBE = model-based estimate; MMRM = mixed model for repeated measures SE = standard error; SF-36v2 = Short-form-36 version 2

Supplementary Figure 6: Incidence of Nausea, Vomiting, Diarrhea, and Constipation Over Time in ATTAIN-1 Participants with BMI of 27 or more and below 30 kg/m^2 and Adiposity-Related Complications

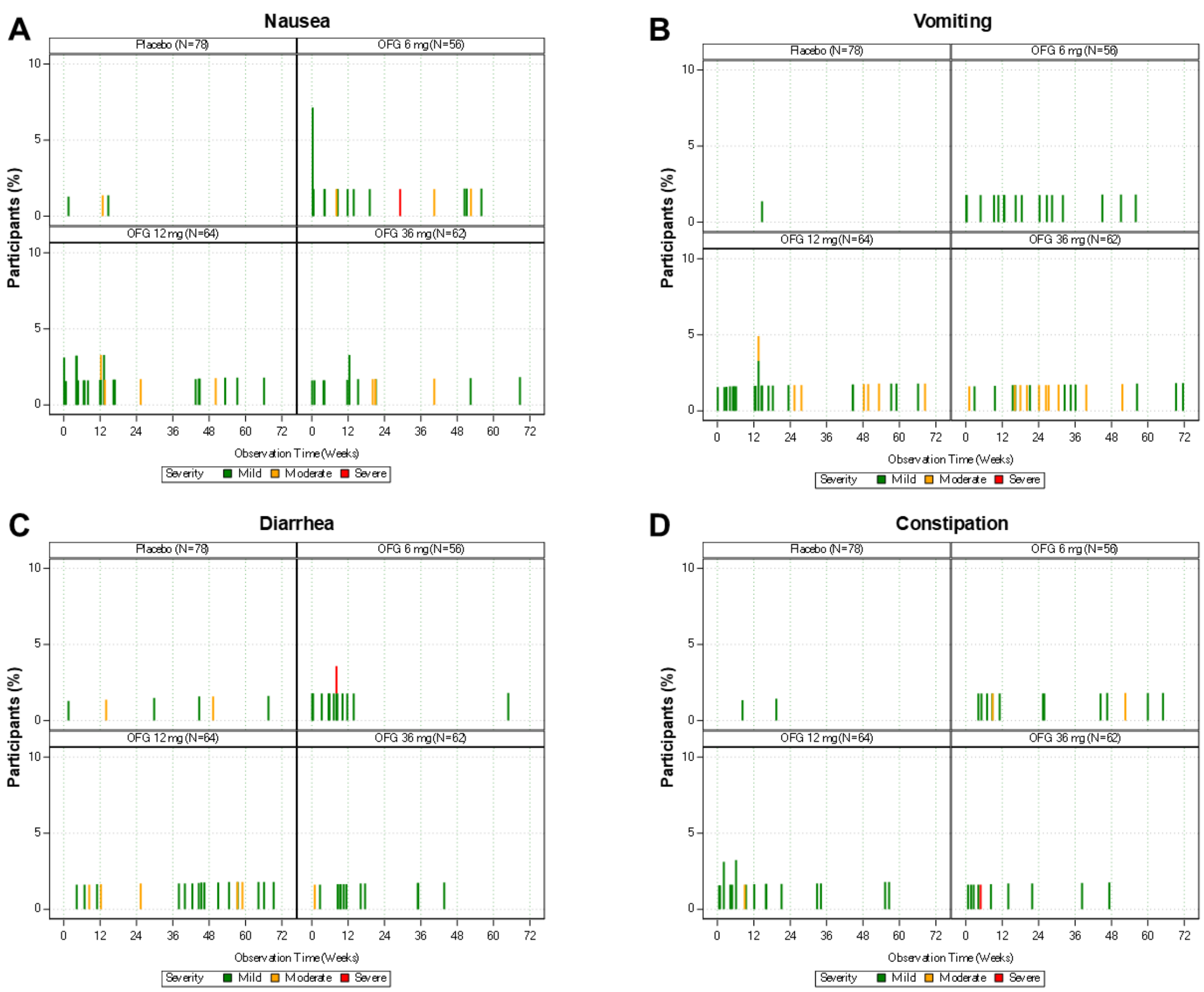

Data are incidence of nausea (A), vomiting (B), diarrhea (C), and constipation (D) as proportions of participants. Incidence refers to the proportion of subjects who have a new event during a time interval.

The counts for the individual GI events may not add up to the combined because subjects may report multiple events during the same time interval.

Vomiting includes preferred terms of vomiting and vomiting projectile; Diarrhea includes preferred terms of diarrhea and frequent bowel movements; Constipation includes preferred terms of constipation, infrequent bowel movements, and feces hard.

MedDRA Version 28.0

Abbreviations: GI = gastrointestinal.

Supplementary Table 1: Country or Region of Origin of ATTAIN-1 Participants with BMI of 27 or more and below 30 kg/m^2 and Adiposity-Related Complications

**Country/Region**	**Orforglipron 5.5 mg N = 56**	**Orforglipron 9 mg N = 64**	**Orforglipron 17.2 mg N = 62**	**Placebo N = 78**
Brazil	4 (7.1)	1 (1.6)	3 (4.8)	4 (5.1)
China	10 (17.9)	12 (18.8)	12 (19.4)	10 (12.8)
India	2 (3.6)	0	0	0
Japan	28 (50.0)	30 (46.9)	31 (50.0)	42 (53.8)
Republic of Korea	2 (3.6)	6 (9.4)	2 (3.2)	6 (7.7)
Slovakia	1 (1.8)	5 (7.8)	5 (8.1)	3 (3.8)
Spain	1 (1.8)	0	1 (1.6)	0
Taiwan	3 (5.4)	1 (1.6)	1 (1.6)	2 (2.6)
United States	5 (8.9)	9 (14.1)	7 (11.3)	11 (14.1)

Data are n (%). N = number of participants in treatment arm; n = number of participants in the respective categories.

Supplementary Table 2: Sex-based Subgroups Baseline Characteristics in ATTAIN-1 Participants with BMI of 27 or more and below 30 kg/m² and Adiposity-Related Complications

	Female Participants				Male Participants			
**Characteristic**	**Orforglipron 5.5 mg N = 31**	**Orforglipron 9 mg N = 29**	**Orforglipron 17.2 mg N = 28**	**Placebo N = 36**	**Orforglipron 5.5 mg N = 25**	**Orforglipron 9 mg N = 35**	**Orforglipron 17.2 mg N = 34**	**Placebo N = 42**
**Age (years)**	47.4 (10.5)	54.2 (9.7)	52.5 (10.1)	54.6 (11.3)	48.2 (14.7)	48.7 (13.1)	47.6 (11.3)	50.6 (12.0)
**Race[a], n (%)**								
Asian	22 (71.0)	20 (69.0)	17 (60.7)	22 (61.1)	23 (92.0)	29 (82.9)	31 (91.2)	39 (92.9)
White	7 (22.6)	8 (27.6)	10 (35.7)	14 (38.9)	1 (4.0)	6 (17.1)	3 (8.8)	2 (4.8)
Black or African American	1 (3.2)	1 (3.4)	0	0	0	0	0	1 (2.4)
Multiple	1 (3.2)	0	1 (3.6)	0	1 (4.0)	0	0	0
**Ethnicity[b], n (%)**								
Hispanic or Latino	5 (16.1)	3 (10.3)	3 (10.7)	5 (13.9)	1 (4.0)	1 (2.9)	0	0
Not Hispanic or Latino	26 (83.9)	26 (89.7)	25 (89.3)	31 (86.1)	24 (96.0)	34 (97.1)	34 (100.0)	42 (100.0)
**Body weight, kg**	74.4 (5.0)	73.7 (7.1)	74.9 (5.6)	73.4 (8.0)	88.2 (6.6)	85.0 (7.7)	84.5 (7.5)	85.4 (7.2)
**BMI, kg/m²**	28.9 (0.8)	28.5 (0.9)	28.5 (0.9)	28.7 (0.8)	28.7 (0.9)	28.5 (0.7)	28.4 (0.8)	28.6 (0.8)
**Waist circumference, cm**	94.1 (6.0)	95.9 (5.6)	97.0 (4.9)	95.6 (7.4)	100.5 (5.9)	99.1 (6.2)	98.8 (4.8)	99.8 (4.9)
**Blood pressure, mm Hg**								
Systolic	117.1 (14.0)	124.3 (11.8)	119.3 (16.5)	121.5 (13.1)	122.6 (11.6)	126.2 (11.8)	120.5 (14.6)	122.5 (10.8)
Diastolic	76.2 (8.9)	80.6 (8.8)	76.7 (9.3)	77.5 (9.2)	78.3 (7.9)	83.3 (9.5)	78.5 (11.6)	82.0 (9.5)
**Pulse rate, bpm**	73.7 (10.2)	74.4 (11.0)	70.1 (8.3)	70.5 (9.3)	72.0 (9.0)	74.1 (10.4)	73.3 (10.6)	73.7 (11.1)
**Lipid parameters, mg/dL[a]**								
Triglycerides	125.7 (48.5)	139.3 (52.3)	118.1 (63.4)	123.3 (50.6)	145.5 (54.9)	141.0 (60.0)	142.8 (45.0)	141.4 (50.5)

Total cholesterol	207.2 (22.8)	219.2 (16.6)	213.0 (19.7)	213.7 (17.8)	183.9 (21.8)	186.4 (18.4)	190.6 (20.3)	198.7 (16.5)
LDL-cholesterol	128.4 (27.0)	126.8 (27.2)	128.7 (25.5)	126.4 (29.8)	103.6 (37.9)	101.9 (42.5)	110.2 (31.1)	119.5 (20.8)
HDL-cholesterol	49.9 (25.3)	57.9 (26.0)	54.3 (25.5)	56.6 (23.1)	44.9 (21.8)	46.8 (17.0)	47.3 (19.0)	46.7 (23.3)
Non-HDL-cholesterol	155.7 (25.8)	157.2 (25.9)	155.0 (27.9)	154.3 (25.2)	136.5 (29.3)	137.5 (25.7)	141.2 (27.2)	149.6 (22.4)
**Lipid parameters, mmol/L[a]**								
Triglycerides	1.4 (48.5)	1.6 (52.3)	1.3 (63.4)	1.4 (50.6)	1.6 (54.9)	1.6 (60.0)	1.6 (45.0)	1.6 (50.5)
Total cholesterol	5.4 (22.8)	5.7 (16.6)	5.5 (19.7)	5.5 (17.8)	4.8 (21.8)	4.8 (18.4)	4.9 (20.3)	5.1 (16.5)
LDL-cholesterol	3.3 (27.0)	3.3 (27.2)	3.3 (25.5)	3.3 (29.8)	2.7 (37.9)	2.6 (42.5)	2.9 (31.1)	3.1 (20.8)
HDL-cholesterol	1.3 (25.3)	1.5 (26.0)	1.4 (25.5)	1.5 (23.1)	1.2 (21.8)	1.2 (17.0)	1.2 (19.0)	1.2 (23.3)
Non-HDL-cholesterol	4.0 (25.8)	4.1 (25.9)	4.0 (27.9)	4.0 (25.2)	3.5 (29.3)	3.6 (25.7)	3.7 (27.2)	3.9 (22.4)
**Hs-CRP[a]**	2.2 (131.4)	1.2 (118.7)	1.4 (117.9)	1.0 (92.7)	1.2 (86.9)	1.0 (119.2)	1.1 (187.8)	1.2 (126.9)
**Prediabetes, n (%)**	8 (25.8)	10 (34.5)	7 (25.0)	15 (41.7)	13 (52.0)	15 (42.9)	13 (38.2)	24 (57.1)
**Comorbidity, n (%)**								
Dyslipidemia	25 (80.6)	22 (75.9)	22 (78.6)	29 (80.6)	18 (72.0)	27 (77.1)	25 (73.5)	30 (71.4)
Hypertension	15 (48.4)	17 (58.6)	12 (42.9)	18 (50.0)	12 (48.0)	18 (51.4)	22 (64.7)	26 (61.9)
Gout/hyperuricemia	1 (3.2)	1 (3.4)	1 (3.6)	1 (2.8)	8 (32.0)	9 (25.7)	9 (26.5)	15 (35.7)
Obstructive sleep apnea	2 (6.5)	0	1 (3.6)	2 (5.6)	4 (16.0)	4 (11.4)	11 (32.4)	7 (16.7)
MASLD[b,c]	6 (19.4)	7 (24.1)	5 (17.9)	10 (27.8)	7 (28.0)	13 (37.1)	9 (26.5)	17 (40.5)

Data are mean (SD) for the given units unless otherwise stated. [a]Race and ethnic groups were reported by the patients. [b]Based on data collected on a prespecified medical history case report form. [c]Based on a Preferred Term search of preexisting conditions as reported by sites.
[a]Baseline lipids and hs-CRP are presented as geometric mean (percentage of CV).
Abbreviations: BMI = body mass index; CV = coefficient of variation; HbA1c = glycated hemoglobin A1c; HDL = high-density lipoprotein; hs-CRP = high-sensitivity C-reactive protein; LDL = low-density lipoprotein; MASLD = metabolic dysfunction-associated steatotic liver disease; N = number of participants in treatment arm; n = number of participants in the respective categories; SD = standard deviation.

Supplementary Table 3: Sex-based Subgroups Analyses of Efficacy Endpoints for the Efficacy Estimand in ATTAIN-1 Participants with BMI of 27 or more and below 30 kg/m^2 and Adiposity-Related Complications

Parameter	Female Participants				Male Participants			
	**Orforglipron 5.5 mg N = 31**	**Orforglipron 9 mg N = 29**	**Orforglipron 17.2 mg N = 28**	**Placebo N = 36**	**Orforglipron 5.5 mg N = 25**	**Orforglipron 9 mg N = 35**	**Orforglipron 17.2 mg N = 34**	**Placebo N = 42**
**Body weight**								
Baseline, kg (SD)	74.4 (5.0)	73.7 (7.1)	74.9 (5.6)	73.4 (8.0)	88.2 (6.6)	85.0 (7.7)	84.5 (7.5)	85.4 (7.2)
Percent change from baseline at Week 72, %	-9.7 (1.3)	-12.5 (1.6)	-18.8 (2.3)	0.9 (0.9)	-4.8 (1.0)	-8.2 (1.0)	-10.0 (1.4)	-1.1 (0.9)
ETD versus Placebo, %, p-Value	-10.6 (-13.7, -7.5), p <0.001	-13.4 (-17.1, -9.8), p <0.001	-19.7 (-24.6, -14.8), p <0.001	-	-3.8 (-6.5, -1.0), p = 0.008	-7.1 (-9.7, -4.5), p <0.001	-8.9 (-12.3, -5.6), p <0.001	-
**Participants reaching 5% body weight reduction**								
Group mean, % (SE)	73.0 (8.0)	71.2 (8.4)	77.9 (8.2)	11.1 (6.4)	58.7 (9.5)	71.7 (8.3)	68.1 (8.5)	20.5 (6.7)
Risk Difference versus placebo, %, p-Value	62.0 (41.4, 82.5), p<0.001	60.2 (39.0, 81.3), p<0.001	66.8 (46.1, 87.6), p<0.001	-	38.2 (15.3, 61.1), p=0.001	51.2 (30.0, 72.4), p<0.001	47.6 (26.6, 68.6), p<0.001	-
**Participants reaching 10% body weight reduction**								
Group mean, % (SE)	36.1 (8.6)	55.9 (9.4)	70.2 (8.8)	2.8 (3.6)	13.1 (6.8)	32.2 (8.5)	59.3 (8.9)	7.9 (4.5)
Risk Difference versus placebo, %, p-Value	33.3 (14.7, 51.9), p<0.001	53.1 (32.9, 73.4), p<0.001	67.4 (48.4, 86.4), p<0.001	-	5.2 (-11.0, 21.4), p=0.527	24.3 (5.2, 43.4), p<0.05	51.3 (31.6, 71.1), p<0.001	-
**Participants reaching 15% body weight reduction**								
Group mean, % (SE)	17.9 (6.7)	38.2 (9.4)	63.2 (8.7)	0.2 (1.0)	4.7 (5.8)	6.1 (4.3)	31.7 (8.2)	2.2 (2.6)
Risk Difference versus placebo, %, p-Value	17.7 (4.2, 31.2), p<0.05	38.0 (19.2, 56.8), p<0.001	63.0 (45.6, 80.4), p<0.001	-	2.5 (-10.3, 15.3), p=0.691	3.8 (-6.6, 14.2), p=0.464	29.4 (12.3, 46.6), p<0.001	-
**BMI, kg/m^2**								
Baseline	28.9 (0.8)	28.5 (0.9)	28.5 (0.9)	28.7 (0.8)	28.7 (0.9)	28.5 (0.7)	28.4 (0.8)	28.6 (0.8)
Change from baseline at Week 72	-2.7 (0.4)	-3.7 (0.5)	-4.9 (0.8)	0.3 (0.3)	-1.2 (0.3)	-2.3 (0.3)	-2.8 (0.5)	-0.3 (0.3)

ETD versus Placebo, p-Value	-3.0 (-3.9, -2.0), p<0.001	-3.9 (-5.0, -2.8), p<0.001	-5.2 (-6.8, -3.5), p<0.001	-	-0.9 (-1.6, -0.1), p = 0.028	-2.0 (-2.8, -1.3), p<0.001	-2.5 (-3.5, -1.4), p<0.001	-
**Participants reaching BMI <25 kg/m²**								
Group mean, % (SE)	18.7 (6.8)	44.5 (9.1)	64.0 (8.3)	2.5 (1.9)	2.7 (3.7)	26.5 (8.0)	50.2 (8.8)	3.5 (3.3)
Risk Difference versus placebo, %, p-Value	16.2 (2.0, 30.4), p = 0.026	42.1 (23.6, 60.5), p <0.001	61.5 (44.8, 78.3), p <0.001	-	-0.7 (-10.9, 9.4), p = 0.888	23.0 (5.6, 40.5), p = 0.010	46.7 (27.9, 65.5), p <0.001	-
**Waist circumference, cm**								
Baseline	94.1 (6.0)	95.9 (5.6)	97.0 (4.9)	95.5 (7.4)	100.5 (5.9)	99.1 (6.2)	98.8 (4.8)	99.8 (4.9)
Change from baseline at Week 72	-7.5 (1.0)	-10.5 (1.4)	-15.4 (1.4)	-1.7 (1.0)	-3.8 (1.0)	-6.9 (0.8)	-6.7 (1.0)	-1.8 (0.8)
ETD versus Placebo, p-Value	-5.9 (-8.6, -3.2), p <0.001	-8.8 (-12.1, -5.5), p <0.001	-13.8 (-17.2, -10.4), p <0.001	–	-2.0 (-4.4, 0.4), p = 0.106	-5.0 (-7.3, -2.8), p <0.001	-4.8 (-7.4, -2.3), p <0.001	–
**Systolic blood pressure, mm Hg**								
Baseline	117.1 (14.0)	124.3 (11.7)	119.3 (16.5)	121.5 (13.1)	122.6 (11.6)	126.2 (11.8)	120.5 (14.6)	122.5 (10.8)
Change from baseline at Week 72	-5.6 (1.7)	-1.1 (2.2)	-5.1 (2.4)	6.5 (1.9)	-7.1 (2.2)	-5.0 (1.8)	-0.6 (2.2)	-1.5 (1.4)
ETD versus Placebo, p-Value	-12.1 (-17.3, -7.0), p <0.001	-7.6 (-13.7, -1.5), p = 0.014	-11.6 (-17.9, -5.4), p <0.001	–	-5.6 (-10.8, -0.4), p = 0.034	-3.5 (-8.0, 1.0), p = 0.127	0.8 (-4.5, 6.1), p = 0.753	–
**Diastolic blood pressure, mm Hg**								
Baseline	76.2 (8.8)	80.6 (8.8)	76.7 (9.3)	77.5 (9.2)	78.3 (7.9)	83.3 (9.5)	78.5 (11.6)	82.0 (9.5)
Change from baseline at Week 72	-2.1 (1.2)	0.5 (1.9)	-2.3 (1.2)	2.7 (1.0)	-3.4 (1.8)	-2.7 (1.5)	0.4 (1.5)	0.2 (1.2)
ETD versus Placebo, p-Value	-4.8 (-7.8, -1.7), p = 0.002	-2.1 (-6.3, 2.0), p = 0.313	-5.0 (-8.1, -1.9), p = 0.002	–	-3.7 (-8.0, 0.6), p = 0.096	-2.9 (-6.7, 0.9), p = 0.131	0.2 (-3.6, 3.9), p = 0.921	–
**Triglycerides**								
Baseline, mg/dL[a]	125.7 (48.5)	139.3 (52.3)	118.1 (63.4)	123.3 (50.6)	145.5 (54.9)	141.0 (60.0)	142.8 (45.0)	141.4 (50.5)
Percent change from baseline at Week 72, %	-20.6 (4.5)	-19.9 (6.0)	-31.5 (5.1)	-8.1 (3.8)	-7.2 (7.4)	-26.8 (6.0)	-28.8 (5.5)	-8.7 (6.2)

ETD versus Placebo, %, p-Value	-13.6 (-24.7, -0.8), p = 0.037	-12.8 (-26.2, 3.1), p = 0.110	-25.4 (-37.0, -11.8), p <0.001	-	1.6 (-17.5, 25.1), p = 0.880	-19.9 (-35.1, -1.2), p = 0.038	-22.1 (-36.8, -4.0), p = 0.019	-
**Total cholesterol**								
Baseline, mg/dL[a]	207.2 (22.8)	219.2 (16.6)	213.0 (19.7)	213.7 (17.8)	183.9 (21.8)	186.4 (18.4)	190.6 (20.3)	198.7 (16.5)
Percent change from baseline at Week 72, %	-4.7 (2.8)	-4.3 (2.3)	-8.1 (3.0)	-1.5 (2.2)	-1.1 (2.8)	-9.0 (3.2)	-4.4 (2.2)	5.5 (2.3)
ETD versus Placebo, %, p-Value	-3.3 (-10.1, 4.0), p = 0.368	-2.9 (-8.7, 3.4), p = 0.362	-6.8 (-13.8, 0.8), p = 0.079	-	-6.3 (-12.7, 0.6), p = 0.074	-13.8 (-20.7, -6.3), p <0.001	-9.4 (-14.8, -3.6), p = 0.002	-
**LDL-C**								
Baseline, mg/dL[a]	128.4 (27.0)	126.8 (27.2)	128.7 (25.5)	126.4 (29.8)	103.6 (37.9)	101.9 (42.5)	110.2 (31.1)	119.5 (20.8)
Percent change from baseline at Week 72, %	-3.1 (4.0)	-7.2 (3.1)	-12.5 (4.4)	-0.4 (3.2)	-0.0 (4.1)	-11.5 (6.2)	-4.8 (4.2)	5.9 (3.7)
ETD versus Placebo, %, p-Value	-2.7 (-12.2, 7.7), p = 0.595	-6.9 (-14.8, 1.9), p = 0.120	-12.2 (-22.2, -0.9), p = 0.036	-	-5.7 (-15.2, 4.9), p = 0.284	-16.4 (-28.9, -1.8), p = 0.029	-10.1 (-19.5, 0.4), p = 0.059	–
**HDL-C**								
Baseline, mg/dL[a]	49.9 (25.3)	57.9 (26.0)	54.3 (25.5)	56.6 (23.1)	44.9 (21.8)	46.8 (17.0)	47.3 (19.0)	46.7 (23.3)
Percent change from baseline at Week 72, %	2.9 (2.9)	10.3 (3.2)	13.1 (3.3)	3.4 (2.2)	1.4 (1.6)	7.3 (2.9)	3.7 (3.5)	7.0 (2.9)
ETD versus Placebo, %, p-Value	-0.5 (-7.3, 6.8), p = 0.893	6.6 (-0.8, 14.6), p = 0.081	9.4 (2.1, 17.3), p = 0.011	-	-5.2 (-11.0, 0.9), p = 0.094	0.3 (-7.0, 8.2), p = 0.944	-3.0 (-10.9, 5.5), p = 0.476	–
**Non-HDL-C[a]**								
Baseline, mg/dL	155.7 (25.8)	157.2 (25.9)	155.0 (27.9)	154.3 (25.2)	136.5 (29.3)	137.5 (25.7)	141.2 (27.2)	149.6 (22.4)
Percent change from baseline at Week 72, %	-6.7 (3.2)	-9.6 (2.6)	-15.9 (4.5)	-3.1 (2.9)	-0.9 (3.6)	-15.1 (5.0)	-7.2 (2.9)	4.8 (2.8)
ETD versus Placebo, %, p-Value	-3.7 (-12.2, 5.5), p = 0.416	-6.7 (-14.0, 1.1), p = 0.090	-13.2 (-23.2, -2.0), p = 0.023	-	-5.4 (-13.5, 3.4), p = 0.220	-19.0 (-29.0, -7.5), p = 0.002	-11.5 (-18.3, -4.1), p = 0.003	–
**Hs-CRP**								
Baseline, mg/L[a]	3.6 (4.5)	1.9 (2.6)	2.2 (2.7)	1.4 (1.3)	1.6 (1.1)	1.5 (1.4)	2.4 (4.1)	2.4 (4.9)

Percent change from baseline at Week 72, %	-43.7 (7.0)	-41.8 (4.7)	-52.4 (8.0)	-5.9 (9.5)	16.2 (24.7)	-23.8 (14.8)	-2.6 (22.6)	-20.8 (13.0)
ETD versus Placebo, %, p-Value	-40.1 (-56.3, -18.0), p = 0.001	-38.1 (-52.1, -20.1), p <0.001	-49.4 (-65.8, -25.4), p <0.001	-	46.8 (-13.8, 149.9), p = 0.158	-3.7 (-41.9, 59.5), p = 0.884	23.0 (-30.0, 116.1), p = 0.471	-

Notes: Baseline data is mean (SD). Change from baseline data is MBE (SE). Data are presented for the efficacy estimand.

[a]Baseline lipids and hs-CRP are presented as geometric mean (percentage of CV). All p-values are nominal as they were not tested for multiplicity.

Abbreviations: BMI = body mass index; CV = coefficient of variation; ETD = estimated treatment difference; HDL = high-density lipoprotein; hs-CRP = high-sensitivity C-reactive protein; LDL = low-density lipoprotein; MBE = model-based estimate; N = number of participants in treatment arm; n = number of participants in the respective categories; SD = standard deviation; SE = standard error

Supplementary Table 4: Efficacy Endpoints for the Treatment Regimen Estimand in ATTAIN-1

Participants with BMI of 27 or more and below 30 kg/m^2 and Adiposity-Related Complications

Parameter	Orforglipron 5.5 mg N=56	Orforglipron 9 mg N=64	Orforglipron 17.2 mg N=62	Placebo N=78
**Body weight**				
Baseline, kg (SD)	80.6 (9.0)	79.9 (9.3)	80.2 (8.2)	79.9 (9.6)
Percent change from baseline at Week 72, %	-6.7 (0.8)	-8.7 (1.1)	-12.3 (1.2)	-0.6 (1.0)
ETD versus Placebo, %, p-Value	-6.1 (-8.6, -3.6), p<0.001	-8.2 (-11.0, -5.3), p<0.001	-11.7 (-14.7, -8.6), p<0.001	-
**Participants reaching 5% body weight reduction**				
Group mean, % (SE)	64.3 (6.2)	67.9 (6.1)	70.0 (6.1)	21.5 (5.3)
Risk Difference versus placebo, %, p-Value	42.8 (26.6, 58.9), p<0.001	46.4 (30.3, 62.4), p<0.001	48.5 (32.2, 64.9), p<0.001	-
**Participants reaching 10% body weight reduction**				
Group mean, % (SE)	20.0 (5.3)	39.9 (6.1)	61.7 (6.4)	9.6 (4.1)
Risk Difference versus placebo, %, p-Value	10.4 (-2.8, 23.6), p = 0.122	30.3 (15.7, 44.8), p<0.001	52.1 (37.0, 67.1), p<0.001	-
**Participants reaching 15% body weight reduction**				
Group mean, % (SE)	8.3 (3.8)	18.2 (4.4)	43.0 (5.9)	2.0 (2.2)
Risk Difference versus placebo, %, p-Value	6.3 (-2.4, 14.9), p = 0.156	16.2 (6.5, 25.9), p = 0.001	41.0 (28.5, 53.5), p<0.001	-
**BMI, kg/m^2**				
Baseline	28.8 (0.8)	28.5 (0.8)	28.4 (0.8)	28.6 (0.8)
Change from baseline at Week 72	-1.7 (0.3)	-2.6 (0.3)	-3.5 (0.4)	-0.1 (0.2)
ETD versus Placebo, p-Value	-1.6 (-2.3, -0.9), p<0.001	-2.5 (-3.2, -1.7), p<0.001	-3.4 (-4.3, -2.5), p<0.001	-
**Participants reaching BMI <25 kg/m^2**				
Group mean, % (SE)	8.8 (3.7)	29.1 (5.6)	55.5 (6.3)	2.3 (1.7)
Risk Difference versus placebo, %, p-Value	6.5 (-1.6, 14.5), p = 0.116	26.8 (15.3, 38.4), p<0.001	53.2 (40.3, 66.2), p<0.001	-
**Waist circumference, cm**				
Baseline	97.0 (6.7)	97.6 (6.1)	98.0 (4.9)	97.8 (6.5)
Change from baseline at Week 72	-5.0 (0.7)	-7.3 (0.9)	-10.3 (0.9)	-1.9 (0.8)
ETD versus Placebo, p-Value	-3.1 (-5.3, -0.9), p = 0.006	-5.4 (-7.9, -2.9), p<0.001	-8.4 (-10.9, -5.9), p<0.001	-
**Systolic blood pressure, mm Hg**				
Baseline	119.6 (13.1)	125.3 (11.7)	119.9 (15.4)	122.0 (11.8)

Change from baseline at Week 72	-6.1 (1.4)	-3.3 (1.5)	-2.5 (1.6)	0.4 (1.3)
ETD versus Placebo, p-Value	-6.5 (-10.2, -2.9), p<0.001	-3.8 (-7.7, 0.2), p = 0.061	-3.0 (-6.9, 0.9), p = 0.134	-
**Diastolic blood pressure, mm Hg**				
Baseline	77.1 (8.4)	82.1 (9.2)	77.7 (10.5)	80.0 (9.6)
Percent change from baseline at Week 72	-2.9 (1.1)	-1.0 (1.2)	-1.1 (1.0)	-0.4 (1.1)
ETD versus Placebo, p-Value	-2.4 (-5.4, 0.5), p = 0.106	-0.5 (-3.6, 2.6), p = 0.740	-0.6 (-3.5, 2.2), p = 0.662	-
**Triglycerides**				
Baseline, mg/dL[a]	134.2 (51.5)	140.2 (56.1)	131.1 (54.3)	132.7 (50.8)
Percent change from baseline at Week 72, %	-16.5 (3.7)	-22.9 (3.9)	-27.3 (3.5)	-8.1 (4.2)
ETD versus Placebo, %, p-Value	-9.1 (-19.6, 2.8), p = 0.127	-16.1 (-26.2, -4.6), p = 0.008	-20.9 (-30.3, -10.2), p<0.001	-
**Total cholesterol**				
Baseline, mg/dL[a]	196.5 (23.0)	200.6 (19.3)	200.4 (20.7)	205.5 (17.4)
Percent change from baseline at Week 72, %	-2.7 (2.1)	-6.7 (1.8)	-5.6 (1.8)	0.6 (1.8)
ETD versus Placebo, %, p-Value	-3.3 (-8.3, 2.0), p = 0.215	-7.3 (-12.0, -2.3), p = 0.005	-6.2 (-10.7, -1.4), p = 0.012	-
**LDL-C**				
Baseline, mg/dL[a]	116.6 (33.9)	112.5 (37.8)	118.2 (29.6)	122.6 (25.3)
Percent change from baseline at Week 72, %	-1.2 (3.2)	-8.5 (3.3)	-7.6 (2.9)	1.7 (2.7)
ETD versus Placebo, %, p-Value	-2.9 (-10.4, 5.3), p = 0.477	-10.0 (-17.3, -2.1), p = 0.014	-9.2 (-16.1, -1.7), p = 0.017	-
**HDL-C**				
Baseline, mg/dL[a]	47.6 (24.2)	51.5 (24.0)	50.4 (23.0)	51.0 (25.1)
Percent change from baseline at Week 72, %	2.0 (1.9)	7.8 (2.2)	8.8 (2.4)	4.1 (2.0)
ETD versus Placebo, %, p-Value	-2.0 (-6.9, 3.3), p = 0.451	3.5 (-2.0, 9.3), p = 0.210	4.5 (-1.3, 10.7), p = 0.132	-
**Non-HDL-C**				
Baseline, mg/dL[a]	146.8 (28.0)	146.1 (26.5)	147.3 (27.7)	151.7 (23.6)
Percent change from baseline at Week 72, %	-3.9 (2.7)	-11.7 (2.4)	-11.4 (2.4)	-0.4 (2.5)
ETD versus Placebo, %, p-Value	-3.5 (-10.3, 3.8), p = 0.334	-11.4 (-17.5, -4.7), p = 0.001	-11.0 (-17.1, -4.5), p = 0.001	-
**Hs-CRP**				
Baseline, mg/L[a]	1.7 (118.3)	1.1 (118.1)	1.2 (154.4)	1.1 (110.9)
Percent change from baseline at Week 72, %	-17.4 (10.1)	-22.4 (10.4)	-32.5 (8.9)	-7.8 (9.7)
ETD versus Placebo, %, p-Value	-10.4 (-34.8, 23.0), p=0.494	-15.8 (-39.6, 17.3), p=0.308	-26.9 (-46.8, 0.7), p=0.055	-
Notes: Baseline data is mean (SD). Change from baseline data is MBE (SE) calculated from ANCOVA. Data are presented for the treatment regimen estimand. Statistical inference for endpoint uses Rubin's				

Rule to combine analyses of imputed datasets. Missing values at the endpoint were imputed using the primary multiple imputation method. Missing data were imputed 50 times.
ANOVA model for baseline measures: Variable = Treatment.
ANCOVA model for endpoint measures: Variable = Baseline + Treatment + Geographic Region + Strata (defined by joint levels of sex [female, male] and prediabetes status [yes, no]) + Treatment*Strata + Treatment*Baseline. All p-values are nominal as they were not tested for multiplicity.
[a]Baseline lipids and hs-CRP are presented as geometric mean (percentage of CV).
Abbreviations: ANCOVA = analysis of covariance; ANOVA = analysis of variance; BMI = body mass index; CV = coefficient of variation; ETD = estimated treatment difference; HDL = high-density lipoprotein; hs-CRP = high-sensitivity C-reactive protein; LDL = low-density lipoprotein; MBE = model-based estimate; N = number of participants in treatment arm; n = number of participants in the respective categories; SD = standard deviation; SE = standard error

.

Supplementary Table 5: Sex-based Subgroups Analyses of Efficacy Endpoints for the Treatment Regimen Estimand in ATTAIN-1

Participants with BMI of 27 or more and below 30 kg/m^2 and Adiposity-Related Complications

Parameter	Female Participants				Male Participants			
	**Orforglipron 5.5 mg N = 31**	**Orforglipron 9 mg N = 29**	**Orforglipron 17.2 mg N = 28**	**Placebo N = 36**	**Orforglipron 5.5 mg N = 25**	**Orforglipron 9 mg N = 35**	**Orforglipron 17.2 mg N = 34**	**Placebo N = 42**
**Body Weight**								
Baseline, kg (SD)	74.4 (5.0)	73.7 (7.1)	74.9 (5.6)	73.4 (8.0)	88.2 (6.6)	85.0 (7.7)	84.5 (7.5)	85.4 (7.2)
Percent change from baseline at Week 72, %	-8.9 (1.3)	-11.2 (1.7)	-16.7 (1.9)	0.6 (1.6)	-4.7 (1.0)	-6.4 (1.4)	-8.6 (1.4)	-1.7 (1.1)
ETD versus Placebo, %, p-Value	-9.5 (-13.6, -5.4), p<0.001	-11.8 (-16.4, -7.3), p<0.001	-17.2 (-22.3, -12.2), p<0.001	-	-3.0 (-5.9, -0.2), p = 0.037	-4.7 (-8.2, -1.2), p = 0.009	-6.9 (-10.5, -3.4), p<0.001	
**Participants reaching 5% body weight reduction**								
Group mean, % (SE)	72.0 (7.9)	66.3 (8.6)	81.0 (8.1)	16.5 (7.6)	59.4 (9.5)	70.3 (8.0)	61.0 (9.0)	26.5 (7.5)
Risk Difference versus placebo, %, p-Value	55.5 (33.7, 77.3), p <0.001	49.8 (26.5, 73.0), p <0.001	64.5 (41.9, 87.1), p <0.001	–	32.9 (9.4, 56.4), p = 0.007	43.8 (22.1, 65.5), p <0.001	34.5 (11.1, 58.0), p = 0.004	–
**Participants reaching 10% body weight reduction**								
Group mean, % (SE)	33.7 (8.4)	52.2 (9.1)	72.8 (8.9)	7.2 (5.7)	7.8 (5.8)	28.9 (8.1)	52.3 (9.0)	11.6 (5.7)
Risk Difference versus placebo, %, p-Value	26.5 (6.2, 46.8), p = 0.011	45.0 (23.6, 66.4), p <0.001	65.6 (44.5, 86.7), p <0.001	–	-3.8 (-19.8, 12.2), p = 0.639	17.4 (-2.3, 37.0), p = 0.083	40.8 (19.5, 62.0), p <0.001	–
**Participants reaching 15% body weight reduction**								
Group mean, % (SE)	17.3 (6.8)	33.6 (8.3)	62.4 (8.7)	1.7 (2.6)	0.2 (1.5)	5.4 (3.6)	26.4 (7.6)	1.9 (2.8)
Risk Difference versus placebo, %, p-Value	15.6 (1.2, 29.9), p = 0.034	31.9 (14.6, 49.2), p <0.001	60.7 (42.6, 78.8), p <0.001	–	-1.7 (-8.4, 5.1), p = 0.617	3.5 (-5.7, 12.6), p = 0.450	24.5 (8.4, 40.6), p = 0.003	–
**BMI, kg/m^2**								
Baseline	28.9 (0.8)	28.5 (0.9)	28.5 (0.9)	28.7 (0.8)	28.7 (0.9)	28.5 (0.7)	28.4 (0.8)	28.6 (0.8)

Change from baseline at Week 72	-2.4 (0.4)	-3.2 (0.5)	-4.6 (0.6)	0.1 (0.3)	-1.0 (0.3)	-1.9 (0.3)	-2.6 (0.5)	-0.3 (0.3)
ETD versus Placebo, p-Value	-2.5 (-3.5, -1.5), p <0.001	-3.3 (-4.5, -2.1), p <0.001	-4.7 (-6.1, -3.4), p <0.001	–	-0.7 (-1.6, 0.2), p = 0.125	-1.7 (-2.6, -0.8), p <0.001	-2.3 (-3.4, -1.2), p <0.001	–
**Participants reaching BMI <25 kg/m²**								
Group mean, % (SE)	20.3 (7.0)	42.1 (8.9)	62.4 (8.8)	3.0 (1.2)	0.0 (0.0)	19.9 (7.3)	47.5 (8.9)	2.0 (2.4)
Risk Difference versus placebo, %, p-Value	17.3 (3.0, 31.6), p = 0.018	39.2 (21.6, 56.8), p<0.001	59.4 (42.1, 76.7), p,0.001	-	-2.0 (-6.7, 2.7), p = 0.407	17.9 (2.7, 33.1), p = 0.021	45.5 (27.3, 63.7), p<0.001	-
**Waist circumference, cm**								
Baseline	94.1 (6.0)	95.9 (5.6)	97.0 (4.9)	95.6 (7.4)	100.5 (5.9)	99.1 (6.2)	98.8 (4.8)	99.8 (4.9)
Change from baseline at Week 72	-7.1 (0.9)	-9.3 (1.3)	-14.5 (1.3)	-2.0 (1.3)	-2.7 (1.1)	-5.6 (1.3)	-6.1 (1.2)	-1.9 (1.1)
ETD versus Placebo, p-Value	-5.1 (-8.4, -1.9), p = 0.002	-7.3 (-11.0, -3.7), p <0.001	-12.5 (-16.1, -8.9), p <0.001	–	-0.9 (-3.9, 2.1), p = 0.570	-3.8 (-7.2, -0.3), p = 0.034	-4.3 (-7.4, -1.1), p = 0.008	–
**Systolic blood pressure, mm Hg**								
Baseline	117.1 (14.0)	124.3 (11.7)	119.3 (16.5)	121.5 (13.1)	122.6 (11.6)	126.2 (11.8)	120.5 (14.6)	122.5 (10.8)
Change from baseline at Week 72	-4.8 (1.7)	-1.4 (2.4)	-4.8 (2.3)	2.7 (2.1)	-7.0 (2.3)	-4.8 (2.0)	-0.6 (2.1)	-1.8 (1.6)
ETD versus Placebo, p-Value	-7.5 (-12.8, -2.2), p = 0.006	-4.2 (-10.5, 2.2), p = 0.194	-7.5 (-13.4, -1.6), p = 0.014	–	-5.2 (-10.6, 0.1), p = 0.056	-2.9 (-7.9, 2.0), p = 0.243	1.2 (-3.9, 6.3), p = 0.636	–
**Diastolic blood pressure, mm Hg**								
Baseline	76.2 (8.8)	80.6 (8.8)	76.7 (9.3)	77.5 (9.2)	78.3 (7.9)	83.3 (9.5)	78.5 (11.6)	82.0 (9.5)
Change from baseline at Week 72	-1.8 (1.3)	0.2 (1.7)	-2.1 (1.3)	0.1 (1.5)	-3.7 (1.8)	-1.9 (1.7)	-0.1 (1.5)	-0.7 (1.4)
ETD versus Placebo, p-Value	-1.9 (-5.9, 2.0), p = 0.334	0.1 (-4.4, 4.6), p = 0.971	-2.3 (-6.3, 1.7), p = 0.264	–	-2.9 (-7.4, 1.6), p = 0.199	-1.2 (-5.5, 3.1), p = 0.575	0.6 (-3.4, 4.5), p = 0.766	–
**Triglycerides**								
Baseline, mg/dL[a]	125.7 (48.5)	139.3 (52.3)	118.1 (63.4)	123.3 (50.6)	145.5 (54.9)	141.0 (60.0)	142.8 (45.0)	141.4 (50.5)
Percent change from baseline at Week 72, %	-19.9 (5.0)	-22.0 (5.4)	-27.8 (4.4)	-7.3 (5.4)	-12.7 (5.3)	-24.7 (5.5)	-27.4 (5.2)	-8.6 (6.2)

ETD versus Placebo, %, p-Value	-13.6 (-26.6, 1.6), p = 0.077	-15.9 (-29.3, 0.1), p = 0.051	-22.1 (-33.7, -8.5), p = 0.003	–	-4.5 (-19.8, 13.7), p = 0.598	-17.6 (-31.7, -0.7), p = 0.042	-20.6 (-34.4, -4.0), p = 0.018	–
**Total cholesterol**								
Baseline, mg/dL[a]	207.2 (22.8)	219.2 (16.6)	213.0 (19.7)	213.7 (17.8)	183.9 (21.8)	186.4 (18.4)	190.6 (20.3)	198.7 (16.5)
Percent change from baseline at Week 72, %	-4.2 (2.8)	-5.7 (2.3)	-6.2 (2.9)	-3.3 (2.7)	-1.1 (3.1)	-7.4 (2.8)	-5.4 (2.0)	4.3 (2.3)
ETD versus Placebo, %, p-Value	-0.9 (-8.3, 7.0), p = 0.808	-2.4 (-9.1, 4.8), p = 0.498	-2.9 (-10.1, 4.9), p = 0.448	–	-5.2 (-12.0, 2.2), p = 0.161	-11.2 (-17.4, -4.5), p = 0.002	-9.3 (-14.6, -3.7), p = 0.002	–
**LDL-C**								
Baseline, mg/dL[a]	128.4 (27.0)	126.8 (27.2)	128.7 (25.5)	126.4 (29.8)	103.6 (37.9)	101.9 (42.5)	110.2 (31.1)	119.5 (20.8)
Percent change from baseline at Week 72, %	-1.9 (4.3)	-8.9 (3.2)	-10.2 (4.3)	-4.5 (3.5)	0.1 (4.8)	-8.5 (5.4)	-5.9 (3.7)	6.9 (3.8)
ETD versus Placebo, %, p-Value	2.7 (-7.8, 14.3), p = 0.629	-4.7 (-13.5, 5.0), p = 0.329	-6.1 (-16.2, 5.2), p = 0.276	–	-6.4 (-16.7, 5.2), p = 0.267	-14.4 (-24.8, -2.5), p = 0.020	-11.9 (-20.6, -2.2), p = 0.018	–
**HDL-C**								
Baseline, mg/dL[a]	49.9 (25.3)	57.9 (26.0)	54.3 (25.5)	56.6 (23.1)	44.9 (21.8)	46.8 (17.0)	47.3 (19.0)	46.7 (23.3)
Percent change from baseline at Week 72, %	2.2 (2.9)	9.9 (3.0)	13.9 (3.6)	1.3 (2.7)	1.7 (2.1)	5.7 (3.2)	4.1 (3.1)	6.9 (2.9)
ETD versus Placebo, %, p-Value	0.9 (-6.5, 8.9), p = 0.816	8.5 (0.7, 16.8), p = 0.032	12.5 (3.7, 22.0), p = 0.005	–	-4.9 (-11.1, 1.9), p = 0.152	-1.0 (-8.6, 7.2), p = 0.795	-2.6 (-10.1, 5.6), p = 0.522	–
**Non-HDL-C**								
Baseline, mg/dL[a]	155.7 (25.8)	157.2 (25.9)	155.0 (27.9)	154.3 (25.2)	136.5 (29.3)	137.5 (25.7)	141.2 (27.2)	149.6 (22.4)
Percent change from baseline at Week 72, %	-5.4 (3.7)	-11.3 (2.7)	-14.0 (4.0)	-5.4 (3.5)	-1.9 (4.0)	-12.1 (4.0)	-9.3 (2.8)	4.2 (3.1)
ETD versus Placebo, %, p-Value	0.1 (-9.7, 10.9), p = 0.993	-6.2 (-14.4, 2.8), p = 0.166	-9.1 (-18.8, 1.8), p = 0.097	–	-5.9 (-14.7, 3.8), p = 0.220	-15.7 (-24.2, -6.3), p = 0.002	-13.0 (-20.0, -5.5), p = 0.001	–
**Hs-CRP**								
Baseline, mg/L[a]	2.2 (131.4)	1.2 (118.7)	1.4 (117.9)	1.0 (92.7)	1.2 (86.9)	1.0 (119.2)	1.1 (187.8)	1.2 (126.9)
Percent change from baseline at Week 72, %	-42.6 (8.3)	-42.1 (5.6)	-51.4 (8.2)	5.4 (14.8)	15.0 (23.2)	4.1 (23.5)	-11.7 (17.7)	-14.7 (13.5)

ETD versus Placebo, %, p-Value	-45.6 (-63.4, -19.0), p = 0.003	-45.1 (-61.0, -22.6), p <0.001	-53.9 (-69.9, -29.3), p <0.001	–	34.8 (-18.3, 122.5), p = 0.241	22.0 (-28.4, 107.7), p = 0.462	3.5 (-35.7, 66.6), p = 0.887	–

Notes: Baseline data is mean (SD). Change from baseline data is MBE (SE) calculated from ANCOVA. Data are presented for the treatment regimen estimand. Statistical inference for endpoint uses Rubin's Rule to combine analyses of imputed datasets. Missing values at the endpoint were imputed using the primary multiple imputation method. Missing data were imputed 50 times.

ANOVA model for baseline measures: Variable = Treatment.

ANCOVA model for endpoint measures: Variable = Baseline + Treatment + Geographic Region 4 + Strata (defined by joint levels of sex [female, male] and prediabetes status [yes, no]) + Treatment*Strata + Treatment*Baseline. All p-values are nominal as they were not tested for multiplicity.

[a]Baseline lipids and hs-CRP are presented as geometric mean (percentage of CV).

Abbreviations: ANCOVA = analysis of covariance; ANOVA = analysis of variance; BMI = body mass index; CV = coefficient of variation; ETD = estimated treatment difference; HDL = high-density lipoprotein; hs-CRP = high-sensitivity C-reactive protein; LDL = low-density lipoprotein; MBE = model-based estimate; N = number of participants in treatment arm; n = number of participants in the respective categories; SD = standard deviation; SE = standard error

Supplementary Table 6. Sex-based Subgroups Analyses of Cardiometabolic Responders Subgroups Analyses in ATTAIN-1 Participants with BMI of 27 or more and below 30 kg/m^2 and Adiposity-Related Complications for the Efficacy Estimand

Parameter	Female Participants				Male Participants			
	Orforglipron 5.5 mg N = 31	Orforglipron 9 mg N = 29	Orforglipron 17.2 mg N = 28	Placebo N = 36	Orforglipron 5.5 mg N = 25	Orforglipron 9 mg N = 35	Orforglipron 17.2 mg N = 34	Placebo N = 42
**HDL target responders (≥50 mg/dL in Females / ≥40 mg/dL in Males)**								
Group mean, % (SE)	71.6 (7.4)	74.2 (6.8)	77.4 (7.6)	67.3 (8.1)	80.5 (4.1)	90.6 (5.1)	77.2 (7.2)	85.5 (5.2)
RD vs. Placebo (95% CI), p-Value	4.33 (-16.5, 25.1), p = 0.679	6.9 (-13.2, 26.9), p = 0.498	10.1 (-11.9, 32.0), p = 0.363	-	-4.9 (-16.0, 6.1), p = 0.377	5.1 (-8.9, 19.1), p = 0.470	-8.2 (-25.3, 8.9), p = 0.342	-
**Triglycerides <150 mg/dL responders**								
Group mean, % (SE)	83.8 (6.6)	63.1 (9.0)	84.7 (6.8)	70.1 (7.6)	65.8 (9.5)	74.8 (7.3)	70.4 (7.8)	61.8 (7.5)
RD vs. Placebo (95% CI)	13.8 (-6.1, 33.6), p = 0.173	-7.0 (-30.0, 16.1), p = 0.555	14.7 (-5.4, 34.7), p = 0.152	-	4.0 (-19.8, 27.7), p = 0.744	13.0 (-7.6, 33.6), p = 0.216	8.5 (-12.7, 29.8) p = 0.431	-
**Waist-to-height ratio <0.53 & BMI <27 kg/m^2 responders**								
Group mean, % (SE)	25.7 (7.2)	41.9 (9.2)	66.6 (8.6)	4.2 (2.2)	31.8 (8.9)	36.0 (8.1)	46.5 (7.7)	10.5 (5.2)
RD vs. Placebo (95% CI), p-Value	21.5 (6.4, 36.6), p = 0.006	37.6 (19.1, 56.2), p < 0.001	62.3 (44.5, 80.2), p <0.001	-	21.3 (0.6, 42.0), p = 0.043	25.5 (6.4, 44.5), p = 0.009	36.0 (17.8, 54.2), p< 0.001	-
**BMI <27 kg/m^2 responders**								
Group mean, % (SE)	57.4 (9.0)	73.3 (7.8)	78.4 (7.4)	10.2 (5.0)	44.3 (10.0)	74.9 (7.9)	69.8 (8.0)	20.3 (6.6)
RD vs. Placebo (95% CI), p-Value	47.2 (26.8, 67.6), p <0.001	63.2 (45.0, 81.3), p <0.001	68.2 (50.8, 85.7), p <0.001	-	24.0 (0.4, 47.6), p = 0.047	54.6 (33.8, 75.4), p <0.001	49.4 (29.4, 69.5), p <0.001	-
**Systolic blood pressure <130 mm Hg and diastolic blood pressure <80 mm Hg responders**								
Group mean, % (SE)	70.8 (7.3)	53.2 (8.8)	70.1 (9.4)	45.0 (8.2)	53.1 (9.3)	57.1 (8.8)	48.0 (8.3)	42.8 (7.7)
RD vs. Placebo (95% CI), p-Value	25.8 (4.9, 46.7), p = 0.016	8.2 (-14.7, 31.1), p =0.476	25.1 (0.8, 49.4), p= 0.043	-	10.3 (-13.2, 33.7), p= 0.387	14.3 (-7.8, 36.5), p= 0.202	5.2 (-16.9, 27.3), p = 0.641	-

Statistical inference for endpoint used Rubin's Rule to combine analyses of imputed datasets. G-computation was used to estimate the unconditional average treatment effect. Group mean, risk difference, CI, and p-value for endpoint measures are from logistic regression model using imputed data. Logistic regression model: Variable = Baseline + Treatment + Geographic Region + Strata (defined by joint levels of sex [female, male] and

prediabetes status [yes, no]) + Treatment*Strata + Treatment*Baseline. All p-values are nominal as they were not tested for multiplicity.
Abbreviations: BMI = body mass index; CI = confidence interval; HDL = high-density lipoprotein; RD = risk difference; SE = standard error

Supplementary Table 7. Cardiometabolic Responders Subgroups Analyses in ATTAIN-1 Participants with BMI of 27 or more and below 30 kg/m^2 and Adiposity-Related Complications for the Treatment Regimen Estimand

Parameter	Orforglipron 5.5 mg N=56	Orforglipron 9 mg N=64	Orforglipron 17.2 mg N=62	Placebo N=78
**HDL target responders (≥50 mg/dL in Females / ≥40 mg/dL in Males)**				
Group mean, % (SE)	77.9 (4.2)	84.1 (4.2)	77.8 (4.8)	77.5 (4.5)
RD vs. Placebo (95% CI), p-Value	0.38 (-10.6, 11.4), p = 0.945	6.57 (-5.6, 18.7), p = 0.287	0.3 (-12.1, 12.6), p = 0.968	-
**Triglycerides <150 mg/dL responders**				
Group mean, % (SE)	76.3 (5.7)	70.3 (5.7)	78.8 (5.2)	66.9 (5.3)
RD vs. Placebo (95% CI)	9.4 (-5.9, 24.6), p = 0.229	3.4 (-12.0, 18.7), p = 0.667	11.9 (-2.7, 26.4), p = 0.111	-
**Waist-to-height ratio <0.53 & BMI <27 kg/m^2 responders**				
Group mean, % (SE)	25.7 (5.4)	34.9 (5.8)	54.2 (5.9)	8.2 (3.4)
RD vs. Placebo (95% CI), p-Value	17.5 (4.9, 30.1), p = 0.007	26.7 (13.6, 39.8), p<0.001	46.0 (32.5, 59.5), p<0.001	-
**BMI <27 kg/m^2 responders**				
Group mean, % (SE)	53.0 (6.8)	66.6 (6.0)	70.3 (5.5)	22.7 (5.6)
RD vs. Placebo (95% CI), p-Value	30.2 (13.0, 47.4), p<0.001	43.8 (27.7, 60.0), p<0.001]	47.6 (31.7, 63.4), p<0.001	-
**Systolic blood pressure <130 mm Hg and diastolic blood pressure <80 mm Hg responders**				
Group mean, % (SE)	64.4 (5.7)	53.2 (6.5)	60.7 (6.0)	46.2 (5.3)
RD vs. Placebo (95% CI), p-Value	18.3 (3.2, 33.3), p = 0.018	7.1 (-9.0, 23.1), p = 0.387	14.6 (-0.9, 30.0), p = 0.064	-

Mean percentage of responders (SE) were based on imputed data and calculated by combining proportion of participants achieving target in imputed data sets using Rubin's rule; SE was calculated in imputed datasets assuming binomial distribution and then combined using Rubin's rule.

Relative risk (95% CI) and p-values were calculated from logistic regression model using imputed data

Data are presented for the treatment regimen estimand. All p-values are nominal as they were not tested for multiplicity.

Abbreviations: BMI = body mass index; CI = confidence interval; HDL = high-density lipoprotein; N = number of participants in treatment arm; n = number of participants in the respective categories; RD = risk difference; SE = standard error.

Supplementary Table 8. Sex-based Subgroups Analyses of Cardiometabolic Responders Subgroups Analyses in ATTAIN-1 Participants with BMI of 27 or more and below 30 kg/m^2 and Adiposity-Related Complications for the Treatment Regimen Estimand

Parameter	Female Participants				Male Participants			
	**Orforglipron 5.5 mg N = 31**	**Orforglipron 9 mg N = 29**	**Orforglipron 17.2 mg N = 28**	**Placebo N = 36**	**Orforglipron 5.5 mg N = 25**	**Orforglipron 9 mg N = 35**	**Orforglipron 17.2 mg N = 34**	**Placebo N = 42**
**HDL target responders (≥50 mg/dL in Females / ≥40 mg/dL in Males)**								
Group mean, % (SE)	71.3 (7.4)	76.6 (6.1)	78.9 (7.1)	67.4 (7.5)	81.7 (2.6)	90.8 (5.1)	75.0 (6.6)	88.2 (4.8)
RD vs. Placebo (95% CI), p-Value	4.0 (-15.8, 23.8), p = 0.691	9.2 (-9.4, 27.9) p = 0.328	11.6 (-8.3, 31.4), p = 0.251	-	-6.5 (-15.3, 2.3), p = 0.146	2.6 (-11.2, 16.4), p = 0.709	-13.2 (-28.4, 2.1), p = 0.090	-
**Triglycerides <150 mg/dL responders**								
Group mean, % (SE)	83.9 (6.6)	65.4 (8.8)	84.9 (6.8)	70.9 (7.6)	66.9 (9.4)	74.3 (7.4)	73.7 (7.5)	63.5 (7.4)
RD vs. Placebo (95% CI), p-value	13.0 (-6.7, 32.7), p = 0.196	-5.5 (-28.3, 17.3), p = 0.636	14.0 (-5.9, 33.9), p = 0.167	–	3.4 (-20.1, 26.9), p = 0.780	10.8 (-9.7, 31.3), p = 0.302	10.2 (-10.6, 30.9), p = 0.336	–
**Waist-to-height ratio <0.53 & BMI <27 kg/m^2 responders**								
Group mean, % (SE)	25.4 (7.0)	39.38 (8.6)	66.5 (8.6)	5.59 (3.6)	26.1 (8.5)	30.4 (8.0)	41.5 (7.6)	12.5 (5.4)
RD vs. Placebo (95% CI), p-Value	19.9 (4.2, 35.7), p = 0.014	33.8 (15.4, 52.2), p< 0.001	61.0 (42.4, 79.5), p <0.001	–	13.6 (-6.5, 33.6), p = 0.183	17.9 (-0.9, 36.7), p = 0.062	29.0 (10.6, 47.4), p = 0.002	–
**BMI <27 kg/m^2 responders**								
Group mean, % (SE)	61.1 (8.8)	67.1 (8.2)	78.5 (7.5)	19.4 (8.1)	46.9 (10.4)	64.0 (8.9)	63.2 (8.0)	26.2 (7.5)
RD vs. Placebo (95% CI), p-Value	41.8 (17.9, 65.6), p<0.001	47.8 (25.1, 70.5), p <0.001)	59.1 (37.4, 80.9), p<0.001	-	20.7 (-4.9, 46.3), p = 0.112	37.7 (14.4, 61.1), p = 0.002	37.0 (15.1, 58.8), p= 0.001	-
**Systolic blood pressure <130 mm Hg and diastolic blood pressure <80 mm Hg responders**								
Group mean, % (SE)	71.5 (7.0)	53.4 (8.5)	70.5 (9.0)	46.0 (7.6)	56.6 (9.4)	51.9 (9.2)	52.0 (8.1)	46.3 (7.5)
RD vs. Placebo (95% CI), p-Value	25.5 (6.3, 44.7), p = 0.010	7.4 (-14.5, 29.3), p = 0.502	24.5 (1.6, 47.4), p = 0.036	–	10.3 (-13.5, 34.2), p = 0.391	5.7 (-17.8, 29.1), p = 0.632	5.8 (-15.0, 26.5), p = 0.583	–

Mean percentage of responders (SE) were based on imputed data and calculated by combining proportion of participants achieving target in imputed data sets using Rubin's rule; SE was calculated in imputed datasets assuming binomial distribution and then combined using Rubin's rule.

Relative risk (95% CI) was calculated from logistic regression model using imputed data
Data are presented for the treatment regimen estimand. All p-values are nominal as they were not tested for multiplicity.
Abbreviations: BMI = body mass index; CI = confidence interval; HDL = high-density lipoprotein; N = number of participants in treatment arm; n = number of participants in the respective categories; RD = risk difference; SE = standard error.

Supplementary Table 9: Patient Reported Outcomes at Week 72 for the Treatment Regimen Estimand in ATTAIN-1 Participants with BMI of 27 or more and below 30 kg/m^2 and Adiposity-Related Complications

Patient-Reported Outcomes, MBE (SE)	Orforglipron 5.5 mg N=56	Orforglipron 9 mg N=64	Orforglipron 17.2 mg N=62	Placebo N=78
**IWQOL – Physical composite**				
Baseline	78.0 (18.9)	77.2 (17.6)	76.3 (19.3)	75.0 (19.0)
Change from baseline	5.3 (2.0)	6.1 (1.8)	6.2 (1.6)	1.0 (2.0)
ETD versus Placebo, MBE (95% CI), p-Value	4.4 (-1.1, 9.8), p = 0.114	5.1 (-0.3, 10.5), p = 0.063	5.2 (0.3, 10.2), p = 0.040	-
**IWQOL – Physical function composite**				
Baseline	78.7 (20.2)	78.5 (19.3)	77.4 (20.3)	77.0 (20.0)
Change from baseline	5.7 (2.1)	6.9 (1.9)	8.0 (1.8)	0.5 (2.0)
ETD versus Placebo, MBE (95% CI), p-Value	5.2 (-0.3, 10.6), p = 0.066	6.3 (1.0, 11.7), p = 0.021	7.5 (2.4, 12.5), p = 0.004	-
**IWQOL – Psychosocial composite**				
Baseline	73.5 (17.6)	70.7 (19.4)	73.1 (18.6)	73.5 (19.8)
Change from baseline	7.1 (1.7)	10.2 (1.9)	12.2 (2.0)	5.1 (1.7)
ETD versus Placebo, MBE (95% CI), p-Value	2.0 (-2.6, 6.6), p = 0.393	5.1 (0.2, 9.9), p = 0.040	7.0 (2.2, 11.9), p = 0.005	-
**IWQOL – Total score**				
Baseline	75.0 (16.5)	73.0 (16.6)	74.2 (17.2)	74.0 (18.3)
Change from baseline	6.5 (1.6)	9.0 (1.7)	10.2 (1.6)	3.7 (1.5)
ETD versus Placebo, MBE (95% CI), p-Value	2.8 (-1.4, 7.1), p = 0.192	5.3 (1.1, 9.5), p = 0.014	6.5 (2.4, 10.6), p = 0.002	-
**SF-36v2 – Physical component score**				
Baseline	54.1 (5.5)	54.5 (3.8)	54.6 (5.6)	53.6 (6.0)
Change from baseline	1.0 (0.7)	0.7 (0.7)	-0.4 (0.8)	-0.1 (0.7)
ETD versus Placebo, MBE (95% CI), p-Value	1.6 (-0.4, 3.5), p = 0.107	1.2 (-0.8, 3.3), p = 0.227	0.2 (-2.0, 2.3), p = 0.875	-
**SF-36v2 – Mental component score**				
Baseline	55.1 (5.5)	54.9 (5.3)	53.0 (6.6)	53.8 (6.1)
Change from baseline	-0.3 (0.9)	-1.0 (0.9)	-0.3 (0.8)	-0.9 (0.8)
ETD versus Placebo, MBE (95% CI), p-Value	-0.6 (-1.8, 2.9), p = 0.630	-0.1 (-2.5, 2.2), p = 0.905	-0.5 (-1.6, 2.7), p = 0.619	-
**SF-36v2 – Physical functioning**				
Baseline	54.2 (5.9)	54.9 (3.4)	54.3 (5.1)	54.1 (5.4)
Change from baseline	1.3 (0.5)	0.7 (0.7)	0.9 (0.7)	-0.9 (0.9)
ETD versus Placebo, MBE (95% CI), p-Value	2.2 (0.2, 4.2), p = 0.031	1.6 (-0.6, 3.8), p = 0.150	1.8 (-0.4, 4.0), p = 0.114	-
**SF-36v2 – Role-physical**				
Baseline	54.6 (4.1)	54.8 (3.9)	54.8 (4.3)	54.6 (5.4)

Change from baseline	-0.1 (0.7)	-0.1 (0.8)	-1.0 (0.9)	-0.6 (0.9)
ETD versus Placebo, MBE (95% CI), p-Value	0.5 (-1.5, 2.5), p = 0.631	0.5 (-1.8, 2.8), p = 0.662	-0.4 (-2.8, 2.0), p = 0.743	-
**SF-36v2 – Bodily pain**				
Baseline	54.1 (8.5)	55.4 (5.7)	55.2 (7.0)	53.7 (8.4)
Change from baseline	-0.4 (1.1)	-0.3 (1.0)	-3.0 (1.2)	-1.1 (1.0)
ETD versus Placebo, MBE (95% CI), p-Value	0.6 (-2.2, 3.5), p = 0.661	0.8 (-1.9, 3.5), p = 0.557	-1.9 (-4.9, 1.0), p = 0.196	-
**SF-36v2 – General health**				
Baseline	54.1 (7.2)	53.4 (6.7)	52.1 (6.7)	52.0 (7.9)
Change from baseline	1.4 (0.8)	1.7 (0.7)	1.9 (0.7)	-0.4 (0.9)
ETD versus Placebo, MBE (95% CI), p-Value	1.9 (-0.4, 4.2), p = 0.106	2.1 (-0.1, 4.3), p = 0.057	2.3 (0.1, 4.5), p = 0.042	-
**SF-36v2 – Vitality**				
Baseline	57.4 (7.4)	57.2 (6.2)	55.7 (8.2)	55.4 (7.7)
Change from baseline	1.4 (0.9)	-1.0 (0.9)	-0.2 (0.9)	-0.7 (1.0)
ETD versus Placebo, MBE (95% CI), p-Value	2.1 (-0.7, 4.8), p = 0.136	-0.3 (-3.0, 2.4), p = 0.833	0.4 (-2.2, 3.1), p = 0.737	-
**SF-36v2 – Social functioning**				
Baseline	55.0 (4.5)	54.1 (5.2)	53.3 (6.0)	53.4 (7.1)
Change from baseline	-0.1 (0.9)	0.1 (1.0)	-0.9 (1.1)	-0.9 (1.2)
ETD versus Placebo, MBE (95% CI), p-Value	0.8 (-2.0, 3.7), p = 0.570	1.0 (-2.0, 3.9), p = 0.514	-0.0 (-3.1, 3.1), p = 0.996	-
**SF-36v2 – Role-emotional**				
Baseline	53.3 (4.3)	53.7 (4.7)	52.9 (5.7)	53.1 (5.2)
Change from baseline	-0.0 (0.8)	-0.1 (0.8)	-0.2 (0.7)	-0.8 (0.9)
ETD versus Placebo, MBE (95% CI), p-Value	0.7 (-1.6, 3.0), p = 0.536	0.7 (-1.7, 3.0), p = 0.562	0.6 (-1.8, 2.9), p = 0.648	-
**SF-36v2 – Mental health**				
Baseline	55.2 (6.3)	55.8 (5.3)	53.2 (6.0)	54.3 (5.6)
Change from baseline	-0.7 (0.8)	-1.2 (1.0)	-0.5 (0.7)	-0.9 (0.8)
ETD versus Placebo, MBE (95% CI), p-Value	0.3 (-2.0, 2.5), p = 0.817	-0.3 (-2.7, 2.2), p = 0.817	0.5 (-1.7, 2.6), p = 0.676	-

Data are presented for the treatment regimen estimand. Baseline data is mean (SD), change from baseline is MBE (SE). All p-values are nominal as they were not tested for multiplicity.
IWQOL-Lite-CT = Impact of weight on quality of life-lite clinical trials; MBE = model-based estimate; N = number of participants in treatment arm; SD = standard deviation; SE = standard error; SF-36 = Short-form-36

Supplementary Table 10: Sex-based Subgroups Analyses of Patient Reported Outcomes at Week 72 for the Treatment Regimen Estimand in ATTAIN-1 Participants with BMI of 27 or more and below 30 kg/m^2 and Adiposity-Related Complications

Parameter	Female Participants				Male Participants			
	Orforglipron 5.5 mg N = 31	Orforglipron 9 mg N = 29	Orforglipron 17.2 mg N = 28	Placebo N = 36	Orforglipron 5.5 mg N = 25	Orforglipron 9 mg N = 35	Orforglipron 17.2 mg N = 34	Placebo N = 42
**IWQOL – Physical composite**								
Baseline	74.8 (20.9)	74.0 (16.4)	66.3 (18.9)	72.0 (20.5)	82.0 (15.5)	79.8 (18.4)	84.5 (15.8)	77.4 (17.5)
Change from baseline	9.9 (2.8)	10.1 (2.8)	7.8 (2.7)	-0.2 (3.0)	1.3 (2.6)	2.8 (2.4)	2.3 (2.0)	2.3 (2.4)
ETD versus Placebo, MBE (95% CI), p-Value	10.1 (2.4, 17.7), p = 0.011	10.3 (2.4, 18.1), p = 0.011	8.0 (0.6, 15.4), p = 0.034	–	-1.0 (-7.8, 5.9), p = 0.783	0.5 (-6.3, 7.3), p = 0.883	0.1 (-6.2, 6.3), p = 0.986	–
**IWQOL – Physical function composite**								
Baseline	74.8 (21.2)	75.5 (18.8)	67.5 (20.9)	73.4 (22.2)	83.4 (18.1)	81.0 (19.6)	85.6 (15.9)	79.9 (17.8)
Change from baseline	10.6 (2.9)	10.7 (3.0)	10.4 (2.8)	0.5 (3.2)	1.4 (2.8)	3.7 (2.4)	3.7 (2.4)	0.7 (2.4)
ETD versus Placebo, MBE (95% CI), p-Value	10.1 (1.9, 18.3), p = 0.016	10.2 (1.7, 18.7), p = 0.019	9.9 (2.4, 17.5), p = 0.011	–	0.7 (-6.4, 7.8), p = 0.849	3.0 (-3.6, 9.5), p = 0.373	3.0 (-3.8, 9.7), p = 0.383	–
**IWQOL – Psychosocial composite**								
Baseline	67.6 (18.0)	63.3 (19.1)	62.7 (18.2)	67.4 (20.2)	80.7 (14.3)	76.9 (17.7)	81.7 (14.1)	78.5 (18.2)
Change from baseline	9.2 (2.6)	15.6 (2.8)	17.4 (2.8)	6.4 (2.7)	5.0 (2.0)	5.5 (2.6)	6.5 (2.5)	3.6 (1.9)
ETD versus Placebo, MBE (95% CI), p-Value	2.9 (-4.2, 10.0), p = 0.419	9.2 (1.9, 16.5), p = 0.014	11.1 (3.7, 18.4), p = 0.004	–	1.4 (-4.0, 6.8), p = 0.614	1.9 (-4.3, 8.0), p = 0.541	2.9 (-2.9, 8.6), p = 0.326	–
**IWQOL – Total score**								
Baseline	70.1 (17.4)	67.1 (15.4)	64.0 (16.9)	69.0 (18.9)	81.2 (13.2)	77.9 (16.2)	82.6 (12.1)	78.1 (17.0)
Change from baseline	9.5 (2.5)	13.8 (2.6)	14.0 (2.4)	4.2 (2.5)	3.8 (2.0)	4.8 (2.2)	5.2 (2.1)	3.1 (1.8)
ETD versus Placebo, MBE (95% CI), p-Value	5.3 (-1.3, 12.0), p = 0.116	9.6 (2.8, 16.5), p = 0.006	9.8 (3.3, 16.3), p = 0.003	–	0.7 (-4.3, 5.8), p = 0.777	1.8 (-3.5, 7.1), p = 0.507	2.2 (-3.0, 7.3), p = 0.402	–
**SF-36v2 – Physical component score**								
Baseline	53.7 (5.8)	54.0 (3.4)	52.9 (6.7)	52.7 (6.8)	54.6 (5.2)	54.9 (4.0)	56.0 (4.0)	54.3 (5.3)

Change from baseline	2.2 (0.9)	1.7 (0.8)	-0.9 (1.3)	-1.5 (1.2)	0.0 (1.1)	-0.1 (1.1)	-0.4 (1.0)	0.2 (0.9)
ETD versus Placebo, MBE (95% CI), p-Value	3.7 (0.7, 6.6), p = 0.016	3.2 (0.2, 6.1), p = 0.035	0.6 (-2.8, 3.9), p = 0.737	–	-0.2 (-2.9, 2.5), p = 0.899	-0.4 (-3.2, 2.5), p = 0.804	-0.6 (-3.3, 2.1), p = 0.644	–
**SF-36v2 – Mental component score**								
Baseline	55.7 (4.8)	56.5 (3.1)	51.4 (7.2)	54.6 (5.8)	54.3 (6.2)	53.6 (6.2)	54.2 (5.9)	53.1 (6.3)
Change from baseline	-1.2 (1.4)	-0.3 (1.0)	-1.4 (1.2)	-0.9 (1.2)	0.6 (1.0)	-1.5 (1.5)	0.7 (1.0)	-0.9 (1.0)
ETD versus Placebo, MBE (95% CI), p-Value	-0.3 (-4.0, 3.3), p = 0.855	0.6 (-2.5, 3.7), p = 0.715	-0.6 (-3.9, 2.8), p = 0.746	–	1.5 (-1.3, 4.3), p = 0.290	-0.6 (-4.0, 2.9), p = 0.745	1.6 (-1.1, 4.4), p = 0.247	–
**SF-36v2 – Physical functioning**								
Baseline	53.3 (7.3)	54.2 (3.5)	52.6 (5.7)	52.9 (6.2)	55.4 (3.1)	55.4 (3.2)	55.7 (4.1)	55.2 (4.3)
Change from baseline	2.6 (0.6)	2.3 (0.9)	1.0 (1.1)	-1.6 (1.4)	0.1 (0.6)	-0.6 (1.1)	0.4 (0.8)	-0.4 (0.9)
ETD versus Placebo, MBE (95% CI), p-Value	4.2 (1.3, 7.2), p = 0.006	4.0 (0.7, 7.2), p = 0.019	2.7 (-1.0, 6.3), p = 0.146	–	0.5 (-1.6, 2.6), p = 0.647	-0.3 (-3.0, 2.4), p = 0.846	0.8 (-1.4, 3.0), p = 0.481	–
**SF-36v2 – Role-physical**								
Baseline	54.7 (3.6)	55.2 (3.4)	53.6 (5.5)	55.0 (3.9)	54.4 (4.8)	54.5 (4.3)	55.9 (2.6)	54.2 (6.4)
Change from baseline	-0.3 (0.9)	0.9 (0.8)	-2.8 (1.5)	-1.2 (1.4)	0.1 (1.0)	-1.0 (1.3)	0.5 (1.0)	-0.1 (1.0)
ETD versus Placebo, MBE (95% CI), p-Value	1.0 (-2.3, 4.3), p = 0.550	2.1 (-1.0, 5.2), p = 0.175	-1.5 (-5.3, 2.3), p = 0.425	–	0.2 (-2.2, 2.6), p = 0.860	-0.8 (-3.8, 2.2), p = 0.579	0.7 (-2.0, 3.3), p = 0.626	–
**SF-36v2 – Bodily pain**								
Baseline	53.9 (9.0)	54.7 (5.4)	52.3 (8.1)	52.6 (8.1)	54.3 (7.9)	56.0 (6.1)	57.6 (5.0)	54.6 (8.7)
Change from baseline	0.7 (1.3)	0.2 (1.5)	-4.3 (1.9)	-2.8 (1.6)	-1.5 (2.0)	-0.4 (1.5)	-2.3 (1.4)	0.4 (1.1)
ETD versus Placebo, MBE (95% CI), p-Value	3.5 (-0.4, 7.3), p = 0.079	2.9 (-1.3, 7.1), p = 0.168	-1.5 (-6.3, 3.3), p = 0.526	–	-2.0 (-6.3, 2.4), p = 0.373	-0.9 (-4.4, 2.7), p = 0.624	-2.7 (-6.1, 0.7), p = 0.116	–
**SF-36v2 – General health**								
Baseline	54.9 (7.2)	54.6 (6.2)	51.8 (7.1)	52.8 (8.5)	53.0 (7.2)	52.3 (7.0)	52.4 (6.5)	51.3 (7.4)
Change from baseline	2.2 (1.1)	1.5 (1.0)	1.5 (1.2)	-0.7 (1.3)	0.8 (1.2)	2.1 (1.1)	1.8 (0.9)	-0.2 (1.1)
ETD versus Placebo, MBE (95% CI), p-Value	2.9 (-0.3, 6.2), p = 0.076	2.3 (-1.0, 5.5), p = 0.169	2.2 (-1.4, 5.7), p = 0.223	–	1.0 (-2.3, 4.3), p = 0.556	2.3 (-0.8, 5.3), p = 0.143	2.1 (-0.7, 4.8), p = 0.141	–

**SF-36v2 – Vitality**								
Baseline	58.2 (7.0)	58.1 (6.1)	52.4 (9.2)	54.5 (7.9)	56.4 (7.9)	56.4 (6.3)	58.4 (6.3)	56.3 (7.5)
Change from baseline	1.3 (1.2)	-0.2 (1.2)	-1.3 (1.4)	-1.0 (1.4)	1.7 (1.4)	-1.5 (1.4)	0.5 (1.2)	-0.5 (1.3)
ETD versus Placebo, MBE (95% CI), p-Value	2.3 (-1.3, 5.9), p = 0.214	0.8 (-2.9, 4.4), p = 0.678	-0.3 (-4.1, 3.4), p = 0.854	–	2.2 (-1.6, 6.0), p = 0.246	-1.0 (-4.8, 2.9), p = 0.619	1.0 (-2.5, 4.5), p = 0.577	–
**SF-36v2 – Social functioning**								
Baseline	53.9 (5.5)	54.5 (5.4)	52.0 (7.1)	53.4 (6.8)	56.3 (2.0)	53.8 (5.1)	54.4 (4.8)	53.4 (7.5)
Change from baseline	-0.4 (1.4)	2.7 (0.9)	-1.5 (1.7)	-0.2 (1.6)	1.5 (1.5)	-2.1 (1.6)	-0.4 (1.4)	-1.5 (1.4)
ETD versus Placebo, MBE (95% CI), p-Value	-0.2 (-4.2, 3.7), p = 0.910	2.9 (-0.4, 6.2), p = 0.085	-1.3 (-5.7, 3.1), p = 0.560	–	3.0 (-0.9, 6.8), p = 0.132	-0.6 (-4.6, 3.4), p = 0.777	1.2 (-2.4, 4.7), p = 0.524	–
**SF-36v2 – Role-emotional**								
Baseline	53.6 (4.2)	55.1 (1.7)	52.1 (7.0)	53.9 (4.1)	53.0 (4.6)	52.6 (5.9)	53.6 (4.2)	52.5 (5.9)
Change from baseline	-0.6 (1.2)	0.2 (1.0)	-1.2 (1.2)	-1.1 (1.4)	0.5 (0.9)	0.0 (1.2)	0.8 (0.9)	-0.5 (1.1)
ETD versus Placebo, MBE (95% CI), p-Value	0.5 (-3.0, 4.0), p = 0.789	1.3 (-2.2, 4.7), p = 0.464	-0.1 (-3.7, 3.5), p = 0.960	–	1.0 (-1.8, 3.7), p = 0.475	0.6 (-2.5, 3.6), p = 0.724	1.4 (-1.5, 4.2), p = 0.344	–
**SF-36v2 – Mental health**								
Baseline	56.1 (5.2)	56.8 (3.7)	51.4 (6.2)	55.0 (4.6)	54.1 (7.4)	55.0 (6.2)	54.7 (5.6)	53.7 (6.3)
Change from baseline	-1.3 (1.2)	-0.3 (1.0)	-1.4 (1.0)	-1.5 (1.2)	-0.2 (1.1)	-1.8 (1.6)	0.5 (1.0)	-0.6 (1.0)
ETD versus Placebo, MBE (95% CI), p-Value	0.2 (-3.2, 3.6), p = 0.896	1.2 (-2.0, 4.3), p = 0.460	0.1 (-3.1, 3.2), p = 0.977	–	0.4 (-2.6, 3.4), p = 0.791	-1.2 (-5.0, 2.5), p = 0.522	1.1 (-1.8, 3.9), p = 0.455	–

Data are presented for the treatment regimen estimand. Baseline data is mean (SD), change from baseline is MBE (SE). All p-values are nominal as they were not tested for multiplicity.IWQOL-Lite-CT = Impact of weight on quality of life-lite clinical trials; MBE = model-based estimate; N = number of participants in treatment arm; SD = standard deviation; SE = standard error; SF-36 = Short-form-36

Supplementary Table 11: Concomitant Antihypertensive Therapy and Lipid-Lowering Therapy in ATTAIN-1 Participants with BMI of 27 or more and below 30 kg/m^2 and Adiposity-Related Complications at Week 72

Parameter	Orforglipron 5.5 mg N=56	Orforglipron 9 mg N=64	Orforglipron 17.2 mg N=62	Placebo N=78
**Concomitant Antihypertensive Therapy**				
No use during trial	36 (64.3)	26 (40.6)	34 (54.8)	34 (43.6)
Use during trial	16 (28.6)	29 (45.3)	18 (29.0)	26 (33.3)
Treatment initiated	0	1 (1.6)	0	0
Increased	0	0	1 (1.6)	4 (5.1)
Not changed	13 (23.2)	23 (35.9)	12 (19.4)	22 (28.2)
Decreased	2 (3.6)	3 (4.7)	2 (3.2)	0
Stopped, no longer needed	1 (1.8)	2 (3.1)	3 (4.8)	0
Cannot be determined	0	0	0	0
Missing	4 (7.1)	9 (14.1)	10 (16.1)	18 (23.1)
**Concomitant Lipid-Lowering Therapy**				
No use during trial	38 (67.9)	31 (48.4)	36 (58.1)	43 (55.1)
Use during trial	14 (25.0)	24 (37.5)	16 (25.8)	17 (21.8)
Treatment initiated	1 (1.8)	0	1 (1.6)	1 (1.3)
Increased	0	1 (1.6)	0	1 (1.3)
Not changed	13 (23.2)	22 (34.4)	11 (17.7)	15 (19.2)
Decreased	0	0	3 (4.8)	0
Stopped, no longer needed	0	1 (1.6)	1 (1.6)	0
Cannot be determined	0	0	0	0
Missing	4 (7.1)	9 (14.1)	10 (16.1)	18 (23.1)

Data are n (%). N = number of participants in treatment arm; n = number of participants in the respective categories.

Supplementary Table 12: Sex-based Subgroups Analyses of Concomitant Antihypertensive Therapy and Lipid-Lowering Therapy in ATTAIN-1 Participants with BMI of 27 or more and below 30 kg/m^2 and Adiposity-Related Complications at Week 72

Parameter	Female Participants				Male Participants			
	Orforglipron 5.5 mg N=31	Orforglipron 9 mg N=29	Orforglipron 17.2 mg N=28	Placebo N=36	Orforglipron 5.5 mg N=25	Orforglipron 9 mg N=35	Orforglipron 17.2 mg N=34	Placebo N=42
**Concomitant Antihypertensive Therapy**								
No use during trial	22 (71.0)	13 (44.8)	17 (60.7)	16 (44.4)	14 (56.0)	13 (37.1)	17 (50.0)	18 (42.9)
Use during trial	9 (29.0)	14 (48.3)	8 (28.6)	8 (22.2)	7 (28.0)	15 (42.9)	10 (29.4)	18 (42.9)
Treatment initiated	0	0	0	0	0	1 (2.9)	0	0
Increased	0	0	0	1 (2.8)	0	0	1 (2.9)	3 (7.1)
Not changed	8 (25.8)	10 (34.5)	5 (17.9)	7 (19.4)	5 (20.0)	13 (37.1)	7 (20.6)	15 (35.7)
Decreased	0	3 (10.3)	1 (3.6)	0	2 (8.0)	0	1 (2.9)	0
Stopped, no longer needed	1 (3.2)	1 (3.4)	2 (7.1)	0	0	1 (2.9)	1 (2.9)	0
Cannot be determined	0	0	0	0	0	0	0	0
Missing	0	2 (6.9)	3 (10.7)	12 (33.3)	4 (16.0)	7 (20.0)	7 (20.6)	6 (14.3)
**Concomitant Lipid-Lowering Therapy**								
No use during trial	26 (83.9)	17 (58.6)	16 (57.1)	14 (38.9)	12 (48.0)	14 (40.0)	20 (58.8)	29 (69.0)
Use during trial	5 (16.1)	10 (34.5)	9 (32.1)	10 (27.8)	9 (36.0)	14 (40.0)	7 (20.6)	7 (16.7)
Treatment initiated	1 (3.2)	0	1 (3.6)	1 (2.8)	0	0	0	0
Increased	0	0	0	1 (2.8)	0	1 (2.9)	0	0
Not changed	4 (12.9)	10 (34.5)	6 (21.4)	8 (22.2)	9 (36.0)	12 (34.3)	5 (14.7)	7 (16.7)
Decreased	0	0	2 (7.1)	0	0	0	1 (2.9)	0
Stopped, no longer needed	0	0	0	0	0	1 (2.9)	1 (2.9)	0
Cannot be determined	0	0	0	0	0	0	0	0
Missing	0	2 (6.9)	3 (10.7)	12 (33.3)	4 (16.0)	7 (20.0)	7 (20.6)	6 (14.3)

Data are n (%). N = number of participants in treatment arm; n = number of participants in the respective categories.

Supplementary Table 13: Summary of Adverse Events by Maximum Severity in ATTAIN-1 Participants with BMI of 27 or more and below 30 kg/m^2 and Adiposity-Related Complications

Parameter, n (%)	Orforglipron 5.5 mg N=56	Orforglipron 9 mg N=64	Orforglipron 17.2 mg N=62	Placebo N=78
**≥1 TEAE**	43 (76.8)	55 (85.9)	49 (79.0)	64 (82.1)
Mild	27 (48.2)	29 (45.3)	30 (48.4)	37 (47.4)
Moderate	13 (23.2)	25 (39.1)	18 (29.0)	22 (28.2)
Severe	3 (5.4)	1 (1.6)	1 (1.6)	5 (6.4)
**Gastrointestinal disorders**	34 (60.7)	41 (64.1)	31 (50.0)	26 (33.3)
Mild	26 (46.4)	29 (45.3)	21 (33.9)	17 (21.8)
Moderate	6 (10.7)	12 (18.8)	9 (14.5)	9 (11.5)
Severe	2 (3.6)	0	1 (1.6)	0
**Metabolism and nutrition disorders**	10 (17.9)	15 (23.4)	9 (14.5)	10 (12.8)
Mild	9 (16.1)	14 (21.9)	9 (14.5)	9 (11.5)
Moderate	0	1 (1.6)	0	1 (1.3)
Severe	1 (1.8)	0	0	0
**Musculoskeletal and connective tissue disorders**	12 (21.4)	9 (14.1)	8 (12.9)	12 (15.4)
Mild	8 (14.3)	4 (6.3)	4 (6.5)	7 (9.0)
Moderate	3 (5.4)	5 (7.8)	4 (6.5)	5 (6.4)
Severe	1 (1.8)	0	0	0
**Infections and infestations**	26 (46.4)	28 (43.8)	22 (35.5)	35 (44.9)
Mild	20 (35.7)	24 (37.5)	14 (22.6)	29 (37.2)
Moderate	6 (10.7)	4 (6.3)	8 (12.9)	6 (7.7)
Severe	0	0	0	0
**Investigations***	8 (14.3)	11 (17.2)	12 (19.4)	15 (19.2)
Mild	7 (12.5)	10 (15.6)	12 (19.4)	15 (19.2)
Moderate	1 (1.8)	1 (1.6)	0	0
Severe	0	0	0	0
**Nervous systems disorders**	9 (16.1)	9 (14.1)	8 (12.9)	3 (3.8)
Mild	4 (7.1)	8 (12.5)	7 (11.3)	3 (3.8)
Moderate	5 (8.9)	1 (1.6)	1 (1.6)	0
Severe	0	0	0	0
**General disorders and administration site conditions**	4 (7.1)	7 (10.9)	7 (11.3)	6 (7.7)
Mild	3 (5.4)	7 (10.9)	4 (6.5)	5 (6.4)
Moderate	1 (1.8)	0	3 (4.8)	1 (1.3)

Severe	0	0	0	0
**Injury, poisoning and procedural complications**	5 (8.9)	6 (9.4)	6 (9.7)	7 (9.0)
Mild	5 (8.9)	6 (9.4)	4 (6.5)	4 (5.1)
Moderate	0	0	2 (3.2)	3 (3.8)
Severe	0	0	0	0
**Respiratory, thoracic and mediastinal disorders**	4 (7.1)	3 (4.7)	5 (8.1)	11 (14.1)
Mild	4 (7.1)	3 (4.7)	4 (6.5)	11 (14.1)
Moderate	0	0	1 (1.6)	0
Severe	0	0	0	0
**Skin and subcutaneous tissue disorders**	6 (10.7)	4 (6.3)	5 (8.1)	7 (9.0)
Mild	6 (10.7)	4 (6.3)	5 (8.1)	4 (5.1)
Moderate	0	0	0	2 (2.6)
Severe	0	0	0	1 (1.3)
**Cardiac disorders**	3 (5.4)	3 (4.7)	2 (3.2)	6 (7.7)
Mild	2 (3.6)	2 (3.1)	2 (3.2)	4 (5.1)
Moderate	0	1 (1.6)	0	1 (1.3)
Severe	1 (1.8)	0	0	1 (1.3)
**Surgical and medical procedures**	4 (7.1)	2 (3.1)	1 (1.6)	6 (7.7)
Mild	3 (5.4)	1 (1.6)	1 (1.6)	5 (6.4)
Moderate	0	1 (1.6)	0	1 (1.3)
Severe	1 (1.8)	0	0	0

Data are n (%). *Including preferred terms such as heart rate increased, aspartate aminotransferase increased, lipase increased. The counts for the individual GI events may not add up to the combined because subjects may report multiple events during the same time interval.
Abbreviations: GI = gastrointestinal; N = number of participants in treatment arm; n = number of participants in the respective categories; TEAE = treatment-emergent adverse event.

Supplementary Table 14: Adverse Events Leading to Study Intervention Discontinuation in ATTAIN-1

Participants with BMI of 27 or more and below 30 kg/m^2 and Adiposity-Related Complications

**Parameter, n (%)**	**Orforglipron 5.5 mg N=56**	**Orforglipron 9 mg N=64**	**Orforglipron 17.2 mg N=62**	**Placebo N=78**
Participants with AEs leading to study intervention discontinuation	1 (1.8)	6 (9.4)	6 (9.7)	5 (6.4)
Gastrointestinal disorders	1 (1.8)	2 (3.1)	4 (6.5)	0
Vascular disorders	0	0	1 (1.6)	0
Hepatobiliary disorders	0	1 (1.6)	0	0
Metabolism and nutrition disorders	0	1 (1.6)	0	0
Investigations	0	0	1 (1.6)	0
General disorders and administration site conditions	0	1 (1.6)	0	1 (1.3)
Musculoskeletal and connective tissue disorders	0	0	0	1 (1.3)
Neoplasms benign, malignant and unspecified	0	1 (1.6)	0	3 (3.8)

Data are n (%). Abbreviation: AE = adverse event; N = number of participants in treatment arm; n = number of participants in the respective categories.

Supplementary Table 15: Vital Signs at Week 72 for the Safety Data Set in ATTAIN-1 Participants with BMI of 27 or more and below 30 kg/m^2 and Adiposity-Related Complications

Parameter	Orforglipron 5.5 mg N=56	Orforglipron 9 mg N=64	Orforglipron 17.2 mg N=62	Placebo N=78
**Pulse (beats/min)**				
Baseline	72.9 (9.6)	74.2 (10.6)	71.9 (9.7)	72.2 (10.4)
Change from baseline at Week 72	5.9 (1.1)	5.8 (1.1)	7.9 (1.1)	1.1 (1.0)
ETD from placebo at Week 72, MBE (95% CI)	4.8 (1.8, 7.8)	4.7 (1.7, 7.7)	6.8 (3.8, 9.8)	-
Change from baseline at Safety Follow-Up	-1.5 (1.3)	1.2 (1.2)	2.0 (1.2)	1.4 (1.3)
ETD from placebo at Safety Follow-Up, MBE (95% CI)	-2.9 (-6.4, 0.7)	-0.2 (-3.7, 3.3)	0.6 (-2.8, 4.1)	-
**Systolic blood pressure, mmHg**				
Baseline (SD)	119.6 (13.1)	125.3 (11.7)	119.9 (15.4)	122.0 (11.8)
Change from baseline at Week 72	-6.1 (1.5)	-3.9 (1.4)	-2.4 (1.4)	1.0 (1.3)
ETD from placebo at Week 72, MBE (95% CI)	-7.1 (-11.0, -3.2)	-4.9 (-8.7, -1.0)	-3.4 (-7.2, 0.5)	-
Change from baseline at Safety Follow-Up	-5.6 (1.5)	-6.5 (1.4)	-3.4 (1.4)	1.6 (1.4)
ETD from placebo at Safety Follow-Up, MBE (95% CI)	-7.1 (-11.2, -3.0)	-8.1 (-12.1, -4.0)	-5.0 (-9.0, -1.0)	-
**Diastolic blood pressure, mmHg**				
Baseline	77.1 (8.4)	82.1 (9.2)	77.7 (10.5)	80.0 (9.6)
Change from baseline at Week 72	-2.8 (1.1)	-1.5 (1.0)	-0.4 (1.0)	0.4 (1.0)
ETD from placebo at Week 72, MBE (95% CI)	-3.2 (-6.0, -0.3)	-1.8 (-4.7, 1.0)	-0.8 (-3.6, 2.0)	-
Change from baseline at Safety Follow-Up	-4.7 (1.1)	-5.4 (1.1)	-2.4 (1.1)	0.9 (1.1)
ETD from placebo at Safety Follow-Up, MBE (95% CI)	-5.6 (-8.7, -2.5)	-6.3 (-9.3, -3.3)	-3.2 (-6.2, -0.3)	-

Data is mean (SD) at baseline and MBE (SE) at Week 72 and SFU. Abbreviations: CI = confidence interval; ETD = estimated treatment difference; MBE = model-based estimate; SD = standard deviation; SFU = safety follow-up; SE = standard error.

Supplementary References

Wharton S, Aronne LJ, Stefanski A, et al. Orforglipron, an Oral Small-Molecule GLP-1 Receptor Agonist for Obesity Treatment. *N Engl J Med*. Nov 6 2025;393(18):1796-1806. doi:10.1056/NEJMoa2511774
